# Heterogeneous PFAS Defluorination and Fluoride Upcycling into Medicinal Agents in Aqueous Electrolytes

**DOI:** 10.1021/acselectrochem.6c00070

**Published:** 2026-05-01

**Authors:** Pramod Tiwari, Nabin Pandey, Soumalya Sinha, Jianbing “Jimmy” Jiang

**Affiliations:** Department of Chemistry, 2514 University of Cincinnati, Cincinnati, Ohio 45221, United States

**Keywords:** PFAS mineralization, Cu electrocatalyst, heterogeneous catalysis, F^−^ upcycling

## Abstract

Per- and polyfluoroalkyl substances (PFASs) mineralization and the upcycling of released fluoride (F^–^) are desired for closing the fluorine cycle. While PFAS filtration and degradation are ongoing research, comprehensive PFAS mineralization followed by immediate valorization of the released F^–^ remains rare. Herein, we report a heterogenized copper­(I) electrocatalyst that fully (100%) decomposes 0.5 mM perfluorooctanoic acid (PFOA), a common groundwater PFAS contaminant, in aqueous KHCO_3_ electrolyte at an anodic current of 5 mA over 18 h. Up to 84% PFOA degradation was achieved at the maximum tested concentration (2 mM) under identical electrochemical conditions, along with a corresponding 91% F^–^ recovery rate. Interestingly, KHCO_3_ acts beyond a supporting electrolyte, influencing the rate-limiting step involved in PFOA defluorination, as revealed by the detection of ^13^CO_2_H-incorporated PFOA under ^13^CO_2_. In addition, we examined the degradation and defluorination rates of 11 PFASs with different chain lengths and configurations, and also valorized the produced F^–^ for the *in-situ* syntheses of medicinally relevant alkyl/arylsulfonyl fluorides (R-SO_2_F, R = −Me, −(CH_2_)_2_Cl, and benzyl), without extracting F^–^ from the post-electrolysis solution and applying current/potential during the fluorination event. This report showcases a rare heterogenized molecular Cu­(I)-electrocatalyst for mineralizing PFASs and the subsequent room temperature F^–^ valorization.

## Graphical abstract

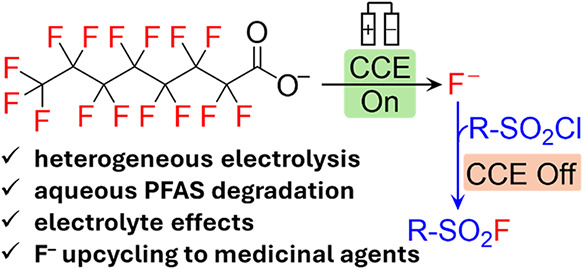

## Introduction

Per- and polyfluoroalkyl substances (PFASs), which are commonly found in commercial and consumer products, are not biodegradable owing to their strong C–F bonds (bond dissociation energies: 106.4–123.4 kcal/mol).^1–4^ Previous reports have shown that PFAS exposure can result in several health issues, including hypercholesterolemia, hormone disruption, and immunosuppression, among others.^5–7^ Unfortunately, 70% of community-based water systems in the USA have tested positive for PFASs, with some above the advisory limit (70 ng/L) according to recent data released by the US Environmental Protection Agency,^8^ thereby affecting 143 million people. Although PFASs can be removed from water by filtration,^9–11^ filtered PFAS waste must be completely mineralized to prevent further environmental exposure. A growing focus on degrading PFASs has led to several strategies for addressing these challenges, including sonochemical,^12–14^ mechanochemical,^15,16^ photochemical,^17–19^ enzymatic,^20,21^ and high-temperature pyrolysis^22^ techniques. However, these methods face substantial challenges, including harsh operating conditions (e.g., toxic chemical oxidants/reductants and high temperature/pressure) and the production of undesired short-chain PFASs from longer ones.^23^

An electrochemical approach for oxidizing PFASs that uses a diverse array of anode materials is of interest because it avoids the use of toxic chemical oxidants and harsh thermal conditions,^24–26^ such materials include boron-doped diamond anodes,^27,28^ SnO_2_/PbO_2_-based anodes,^29^ and Ce-doped porous nanocrystalline PbO_2_ film electrodes.^30^ However, most of these electrode materials require high applied currents (50–380 mA) to achieve high PFAS degradation rates.^31^ Other common drawbacks include electrode-material costs, electrode-preparation complexities, and toxic ions leaching from the electrode.^31^ Therefore, the use of inexpensive and non-toxic electrode materials is highly desirable for mineralizing PFASs into more benign products, such as fluoride ions (F^–^), under ambient conditions. In addition, valorizing the F^–^ produced during PFAS mineralization has rarely been reported.^32^

With the exception of **[CuT2]** ^**+**^, a Cu­(I) catalyst recently reported by us (Figure 1A),^33^ molecular electrocatalysts for selectively degrading PFASs under ambient conditions are rare. **[CuT2]** ^**+**^ promotes the degradation of perfluorooctanoic acid (PFOA), the most abundant PFAS detected in groundwater, by 93% under homogeneous conditions in an acetonitrile electrolyte when a reductive current of 5 mA is applied.^33^ However, the development of catalytic materials by incorporating molecular catalysts into solid electrode materials is more promising as it minimizes the required amount of catalyst, and contributes to improving the scalability of the system for PFAS remediation. The aqueous medium is a desired solvent choice for PFAS remediation to avoid any environmental concerns and practical limitation.^34^ In this study, the **[CuT2]** ^**+**^ catalyst was immobilized on the surface of carbon paper electrodes, which enables its application in aqueous media relevant for real-world PFAS treatment. Inspired by progress in materials science for the anodic oxidation of PFOA, we developed carbon electrode materials for oxidatively mineralizing PFASs by drop-casting **[CuT2]** ^**+**^ onto carbon paper electrodes. Such heterogenized **[CuT2]** ^**+**^ catalysts were found to decompose PFOA by up to 84% in an aqueous KHCO_3_ electrolyte when an anodic current of 5 mA was applied over 18 h, with 91% of the F^–^ recovered following electrolysis, underscoring its ability to efficiently mineralize PFOA in the aqueous electrolyte. Drinking water and other environmental samples generally contain low PFAS concentrations, which can be separated and collected to form a concentrated wastewater stream. The **[CuT2]** ^**+**^-drop-cast carbon paper anode electrode was specifically designed to degrade PFOA captured and concentrated from drinking water and soil, which is achieved following a reported procedure.^35,36^ Comprehensive mineralization of the PFAS in the wastewater stream under ambient conditions is still not well established. In this report, we aim to develop molecular Cu catalyst immobilized electrode materials to degrade the concentrated PFAS waste in the aqueous media. We also extended the applicability of the **[CuT2]** ^**+**^-drop-cast carbon electrode to the degradation of three additional major classes of PFAS commonly found in the environment: perfluoroalkyl carboxylic acids (PFCAs), perfluorodicarboxylic acids (PFDCAs), and fluorotelomeric carboxylic acids (FTCAs).^37,38^ In addition, we studied the electrocatalytic PFAS degradation activities of the heterogenized **[CuT2]** ^**+**^ catalyst at different applied currents and PFAS concentrations, and in various supporting electrolytes. Interestingly, aqueous KHCO_3_ did not serve as an inert supporting electrolyte for electrochemical PFAS oxidation; rather it was found to influence the rate of PFAS decarboxylation. We also detected only trace amounts of some short-chain PFAS products by electrospray ionization mass spectrometry (ESI-MS), consistent with our previously reported homogeneous system that uses the identical Cu­(I) catalyst but in molecular form, which did not yield any short-chain products. These results suggest that immobilizing **[CuT2]** ^**+**^ onto a carbon electrode does not alter the efficiency of the selective PFAS-degradation process, thereby providing a rare example of a molecular-catalyst-immobilized electrode material for the ambient degradation of PFAS.

**Figure 1:**
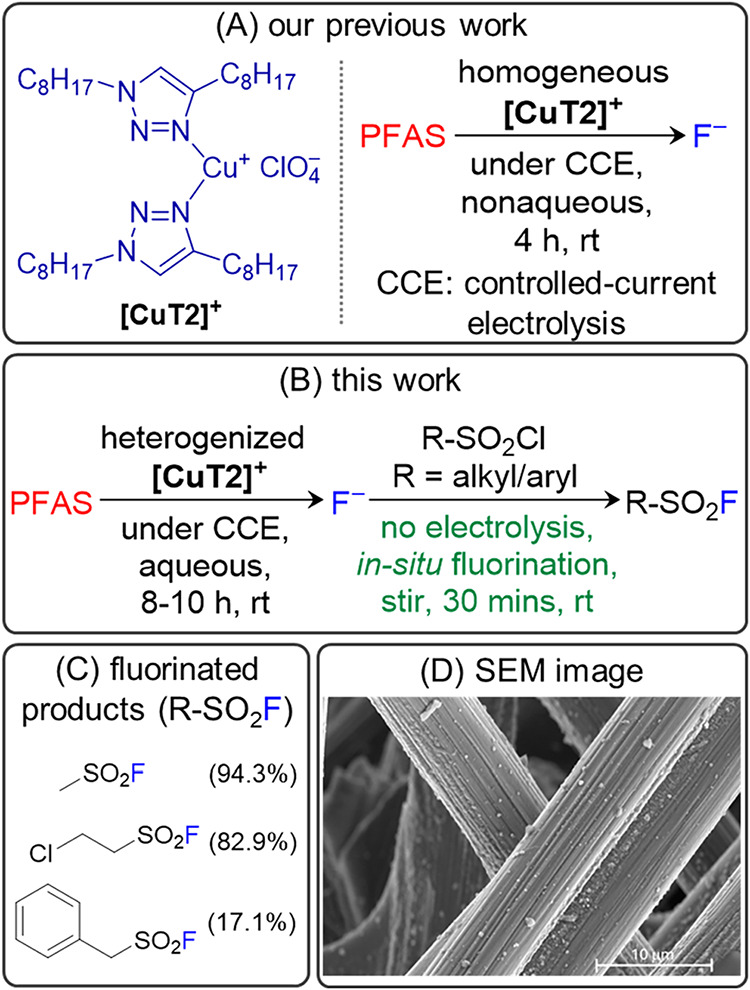
(A) Our previous work that focused on homogeneous PFAS degradation with a molecular Cu­(I) electrocatalyst in a non-aqueous electrolyte under controlled-current electrolysis (CCE) conditions. (B) Heterogeneous PFAS degradation electrocatalyzed by a **[CuT2]** ^**+**^-immobilized carbon paper electrode, followed by *in-situ* fluorination of an alkyl/aryl sulfonyl chloride (R-SO_2_Cl) in the absence of an applied current (this study). (C) Fluorinated products and associated yields following the scheme shown in panel (B). (D) Scanning electron microscopy (SEM) image of a **[CuT2]** ^**+**^-adsorbed carbon electrode surface (the SEM image of bare carbon paper is shown in Figure S1).

Furthermore, we valorized the F^–^ produced during heterogeneous PFAS mineralization for the *in-situ* synthesis of methylsulfonyl fluoride (MeSO_2_F) in the absence of an applied current. MeSO_2_F is of particular medicinal interest as an irreversible inhibitor of human cholinesterase, a target relevant to the treatment of neurological disorders, including Alzheimer’s disease and myasthenia gravis.^39^ Harnessing F^–^ for the synthesis of MeSO_2_F without extracting it from the post-PFOA-degraded electrolyte enables direct access to free F^–^ by avoiding multistep filtration or purification processes (Figure 1B,1C). We also evaluated scope and selectivity by fluorinating chloroethylsulfonyl chloride (Cl­(CH_2_)_2_SO_2_Cl) and benzylsulfonyl chloride (BzSO_2_Cl), which simply involved stirring the alkyl/aryl sulfonyl chloride in a solution of the F^–^ produced during heterogeneous PFAS mineralization.

## Results and Discussion

The synthesis of **[CuT2]** ^**+**^ and its drop-casting onto carbon electrodes were performed according to our previously reported procedures (see the Experimental Section for further experimental details).^33,40^ Scanning electron microscopy (SEM) (Figure 1D) and energy-dispersive X-ray analysis (Figure S2) confirmed that **[CuT2]** ^**+**^ had been deposited on the bare carbon paper electrode (Figure S3). Our previous research efforts revealed that the **[CuT2]** ^**+**^ **-**immobilized carbon electrode is robust for more than 14 h in aqueous media during the electrocatalytic reduction of CH_2_Cl_2_ to hydrocarbons.^40^ Inspired by our previous studies, we examined the performance of the heterogenized **[CuT2]** ^**+**^ catalyst during the decomposition of PFAS under electrochemical oxidation conditions in an aqueous electrolyte.

### PFOA Mineralization Using the Heterogenized [CuT2]^+^ Catalyst at 5 mA

We examined the oxidation of PFOA over the **[CuT2]** ^**+**^-immobilized carbon anode using controlled-current electrolysis (CCE) in a N_2_-saturated 0.1 M KHCO_3_ solution containing 2 mM PFOA in the anodic compartment of a membrane-separated H-cell. Gaseous products were detected by constantly monitoring the headspace of the anodic compartment during electrolysis using gas chromatography (GC), and the anolyte solution from the same compartment was systematically withdrawn every 3 h to detect products using ^19^F nuclear magnetic resonance (NMR) spectroscopy and ion chromatography (IC) (see Experimental Section for details). GC revealed that CO_2_ is immediately evolved when an anodic current of 5 mA is applied (Figure S4), as expected for the electrochemical decarboxylation of PFOA.^41,42^ The potential at the working electrode, which was initially ∼2.1 V vs. Ag/AgCl, was observed to increase to more positive potentials as electrolysis progressed (Figure S5). Time-dependent ^19^F NMR spectra collected during CCE revealed that a new peak appeared at −119 ppm over 18 h, suggestive of the formation of F^–^ (Figure 2A), which was confirmed by recording the spectrum of a standard sample of KF in PFOA-free aqueous electrolyte solution (Figure S6). Furthermore, time-dependent ^19^F NMR data revealed a noticeable decrease in the PFOA concentration in the anolyte solution during 18 h of CCE at a current of 5 mA (Figure 2B), with 84.2% PFOA degradation determined by adding an internal standard (KPF_6_) to the NMR sample (Figures 2B and S7). Anolyte solutions collected at 3 h intervals during CCE were also used to record time-dependent IC data; strong F^–^ signals were observed (Figure S8), thereby further confirming that F^–^ is formed during PFOA oxidation. Approximately 91% free F^–^ was recovered from the post-CCE aqueous anolyte after 18 h of CCE using a **[CuT2]** ^**+**^-adsorbed carbon anode (Figure 2B), based on the IC calibration curve generated using standard samples of KF in deionized water (Figure S9). The CCE experiment was performed under identical electrochemical conditions for 10 h, and the anolyte solution was stirred for another 8 h in the absence of any applied current to examine the self-degradation of PFOA during the oxidation event. Time-dependent ^19^F NMR spectroscopy revealed that the collected anolyte solutions exhibited only 5% change in PFOA concentration after stopping CCE, which suggests that PFOA degradation requires a continuous application of current with **[CuT2]** ^**+**^ deposited carbon electrode (Figure S10). As a control experiment, a **[CuT2]** ^**+**^-drop-cast carbon paper electrode was placed in the same setup used to electrochemically oxidize PFOA for 18 h, but in the absence of applied current. Analysis of the post-anolyte solution by IC revealed a lack of F^–^, while ^19^F NMR spectroscopy revealed an 8% degradation rate, which is possibly ascribable to PFOA adsorption on the electrode surface (Figures S11 and S12). Hence, PFOA mineralization requires the application of a current during electrochemical oxidation throughout the 18 h of CCE. The overall rate of the heterogenized-**[CuT2]** ^**+**^-catalyst-promoted PFOA degradation event was also calculated under these electrochemical conditions by plotting the logarithm of the ratio of the PFOA concentration to the initial PFOA concentration (2 mM) as a function of time during CCE; the linear relationship is indicative of pseudo-first-order reaction kinetics with a rate constant of 0.085 h^–1^ (Figure 2C), which is comparable to the reported rate for the electrochemical oxidation of PFOA promoted by a SnO_2_/PbO_2_-based anode.^29^

**Figure 2:**
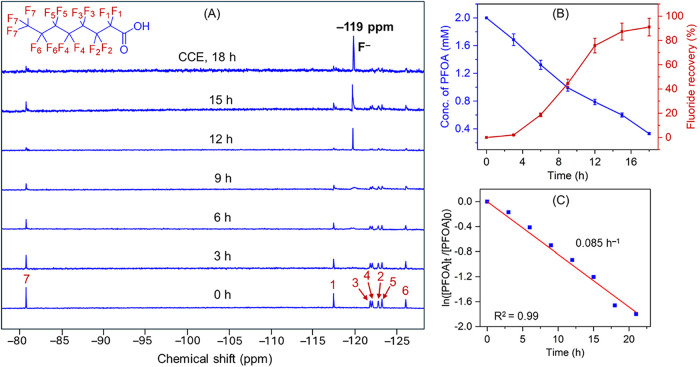
(A) Time-dependent ^19^F NMR spectra recorded for an aqueous electrolyte solution containing 2 mM PFOA before and during CCE at an anodic current of 5 mA. (B) Calculated PFOA concentration in the electrolyte solution and percentage of free F^–^ recovered as functions of time over 18 h under electrolysis conditions. (C) Logarithmic ratio of the PFOA concentration ([PFOA]_t_) to the initial PFOA concentration ([PFOA]_0_) as a function of time.

To confirm that **[CuT2]** ^**+**^ is essential for delivering high PFOA degradation and defluorination rates, we subjected 2 mM PFOA to CCE at 5 mA using a bare carbon paper working electrode; however, only a 10% PFOA-decomposition rate was observed (Figure S13). A control experiment using a CuO-deposited electrode resulted in only 26% PFOA degradation and 7% defluorination, confirming that the **[CuT2]** ^**+**^ catalyst is the active species that promotes PFOA degradation (Figures S14 and S15). CCE was performed under identical conditions in another control experiment with the heterogenized **[CuT2]** ^**+**^ catalyst but without PFOA in the aqueous electrolyte. The post-CCE anolyte solutions collected from these control experiments (without catalyst or PFOA) did not show any F^–^ by IC (Figures S16 and S17). Taken together, these results confirm that the F^–^ signal detected by IC is due to PFOA mineralization promoted by the heterogenized **[CuT2]** ^**+**^ catalyst. We also conducted a control experiment to determine whether or not the Nafion binder degrades and releases F^–^ during electrochemical oxidation. For this purpose, a carbon paper working electrode was prepared by drop-casting 100 μL of a Nafion solution devoid of **[CuT2]** ^**+**^ (95 vol % propanol and 5 vol % Nafion dispersion). Electrochemical oxidation at 5 mA, both in the presence and absence of PFOA, was performed heterogeneously under CCE conditions in 0.1 M KHCO_3_ in a N_2_-sparged H-cell. The post-CCE anolyte solution did not show any F^–^ by IC, confirming that the detected F^–^ is ascribable to PFOA mineralization catalyzed by **[CuT2]** ^**+**^ rather than the degradation of the Nafion binder. The electrode potentials of the **[CuT2]** ^**+**^-immobilized and Nafion-immobilized carbon electrodes were compared, which revealed that the anodic PFOA-oxidation potential recorded using the heterogenized **[CuT2]** ^**+**^ catalyst during CCE shifted more than that of the Nafion-immobilized carbon electrode (Figure S18). This comparison confirmed that the stronger shift in anodic potential is due to the catalytic activity of the **[CuT2]** ^**+**^ catalyst on the carbon electrode surface rather than that of the Nafion binder.

### PFAS Degradation and Free F^-^ Recovery According to Anodic Current

We previously studied PFOA degradation catalyzed by homogeneous **[CuT2]** ^**+**^ in a non-aqueous electrolyte, which showed that the PFOA degradation rate increased with increasing applied cathodic current.^33^ To examine similar effects operating in the current heterogeneous study under electro-oxidation conditions, we performed CCE at three anodic currents (5, 7.5, and 10 mA) for 8 h using the same **[CuT2]** ^**+**^-drop-cast carbon anode in N_2_-saturated 0.1 M KHCO_3_ containing 2 mM PFOA, with PFOA-degradation and free-F^–^-recovery rates shown in Figure 3A and listed in Table 1. In brief, both PFOA degradation and F^–^ recovery were observed to increase from 53 to 82.3% and 50 to 89%, respectively, when the applied current was increased from 5 to 10 mA under the same CCE conditions. These results indicate that increasing the applied current also enhances the degree of PFOA decomposition (see Figures S19–S21 for all potential vs. time profiles, ^19^F NMR spectra, and IC data related to these CCE experiments). Furthermore, we calculated the Coulombic efficiency (CE) for the defluorination process at three different oxidatively applied currents; values of 2.7, 3.8, and 3.75% were recorded at 5, 7.5, and 10 mA, respectively. These CEs are 150-times higher than those previously reported for oxidative electrochemical PFOA degradation in aqueous media.^43^

**Figure 3:**
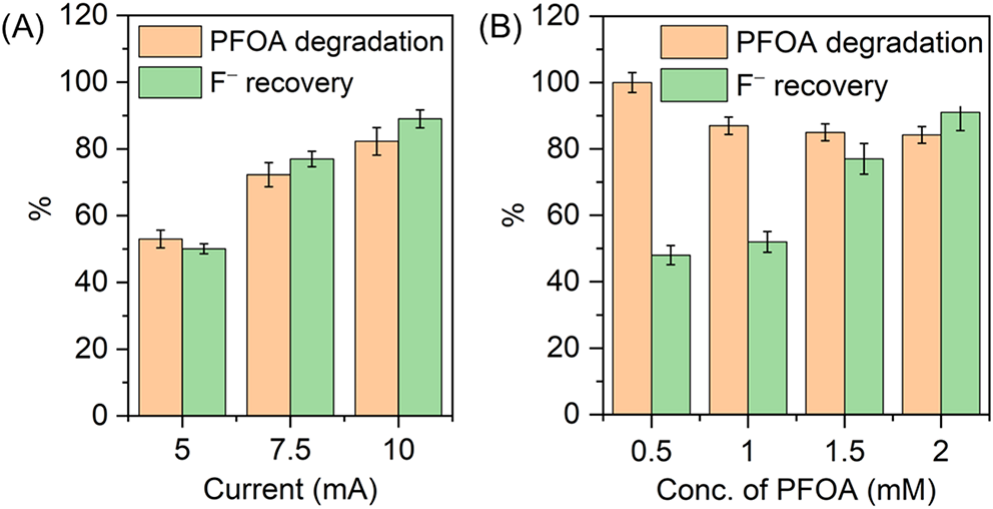
%PFOA degradation and %fluoride recovery. (A) At three different oxidative currents, 5, 7.5, and 10 mA (PFOA concentration = 2 mM) and (B) At four different PFOA concentrations, 0.5, 1.0, 1.5, and 2.0 mM (applied current = 5 mA).

**Table 1:** PFOA-Degradation and Fluoride-Recovery Rates at Various Currents, CCE Durations, and PFOA Concentrations

initial PFOA concentration (mM)	applied current (mA)	CCE duration (h)	PFOA degradation (%)	fluoride recovery (%)
2.0	5.0	8	53.0	50
2.0	7.5	8	72.3	77
2.0	10.0	8	82.3	89
2.0	5.0	18	84.2	91
1.5	5.0	18	85.0	77
1.0	5.0	18	87.0	52
0.5	5.0	18	100	48

### Effect of Initial PFOA Concentration on Degradation

We investigated the relationship between PFOA degradation and the initial PFOA concentration in the aqueous electrolyte under CCE conditions by applying an anodic current of 5 mA for 18 h in 0.1 M KHCO_3_ solutions with different initial PFOA concentrations (0.5, 1.0, 1.5, and 2 mM; Figures S22–S27). PFOA was highly degraded (∼100%) when an initial PFOA concentration of 0.5 mM was used (Figure 3B), but comparatively low degradation rates were recorded (80 ± 4%, Table 1) at initial PFOA concentrations greater than 0.5 mM. The lower levels of PFOA degradation observed at higher PFOA concentrations can be explained by the formation of short-chain PFAS intermediates, as evidenced by CCE (*vide infra*). These short-chain PFAS intermediates compete with the parent PFAS at the active sites of the electrode, resulting in mass-transport issues for the parent PFAS chains across the diffusion-layer interface between the electrode surface and bulk solution.^44^ The low fluoride recovery could be due to incomplete mineralization of PFOA to F^–^.

### Detecting by-Products During PFOA Degradation

By-products from the degradation of PFOA (other than free F^–^) were detected by subjecting aliquots collected from the post-CCE solutions to ESI-MS. CCE was performed at 5 mA for 18 h using the **[CuT2]** ^**+**^-immobilized carbon electrode in 0.1 M KHCO_3_ containing 2 mM PFOA. Negative mode ESI-MS subsequently revealed signals at m/z values of 412.96, 362.96, 312.97, 262.97, 212.97, 318.97, 268.98, 218.98, and 168.98 that correspond to C_7_F_15_CO_2_^–^, C_6_F_13_CO_2_^–^, C_5_F_11_CO_2_^–^, C_4_F_9_CO_2_^–^, C_3_F_7_CO_2_^–^, C_6_F_13_^–^, C_5_F_11_^–^, C_4_F_9_^–^, and C_3_F_7_^–^, respectively (Figure S28). In addition, we observed a signal at m/z 237.00 by positive mode ESI-MS that may correspond to the [M + H]^+^ peak of CF_3_(CF_2_)_3_OH (235.99) in the aqueous medium. These signals were not detected by ESI-MS when pre-CCE solutions containing PFOA were analyzed (Figure S29), which confirms that these organofluorine side products are PFOA-degradation products and not due to the uncontrolled fragmentation of PFOA during ESI-MS. However, the ^19^F NMR spectra of the post-CCE solutions did not show any signals corresponding to organofluorine products, which suggests that insubstantial yields of these organofluorine compounds are produced during PFOA degradation.

### Integrity of the Heterogenized Catalyst

The **[CuT2]** ^**+**^-coated carbon paper working electrode was examined by X-ray photoelectron spectroscopy (XPS) to evaluate the structural integrity of the heterogenized catalyst coating. XPS survey spectra of the electrode before and after electrolysis (conditions: CCE at 5 mA, 18 h, 2 mM PFOA) revealed two peaks at binding energies of 932.7 and 952.8 eV ascribable to Cu 2p_3/2_ and Cu 2p_1/2_, respectively (Figure 4A,4B),^40^ consistent with the presence of the Cu catalyst throughout the electrolysis process during PFOA degradation (Table S1). Furthermore, elemental copper on the surface of the carbon paper working electrode was examined by XPS after rinsing with ethanol to remove any residual catalyst. The resulting spectra closely resemble those of the carbon paper electrode coated only with the Nafion binder, which reveals that no copper was electrodeposited on the surface of the carbon paper (Figures S30 and S31). The leaching of catalyst from the electrode surface was also tested by using inductively coupled plasma mass spectrometry (ICP-MS) upon constructing the calibration curve (Figure S32). Accounting for the amount of copper obtained based on ICP-MS data, 4.62% of the **[CuT2]** ^**+**^ catalyst was estimated to be leached out from the carbon paper working electrode surface after CCE in the presence of PFOA under identical electrochemical conditions, suggesting high stability of the molecular Cu catalyst on the carbon paper electrode during the CCE.

**Figure 4:**
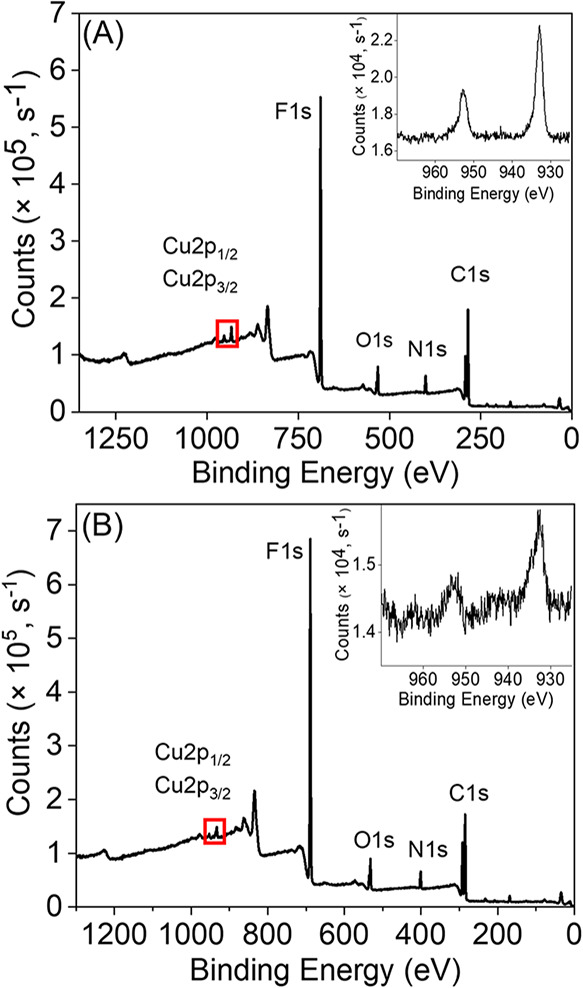
X-ray photoelectron spectra of the heterogenized Cu­(I) electrocatalyst. (A) Before and (B) after electrolysis (conditions: CCE at 5 mA, 18 h, 2 mM PFOA). Insets: Expanded Cu 2p_3/2_ and Cu 2p_1/2_ region.

### Effect of the Supporting Electrolyte on the Electrochemical PFOA Oxidation Process

PFAS is typically deprotonated (Figure 5A) and then anodically oxidized to produce CF_3_(CF_2_)_n_CO_2_^•^ radicals during electrochemical oxidation in an aqueous electrolyte.^44,45^ Subsequent decarboxylation of the CF_3_(CF_2_)_n_CO_2_^•^ radical (Figure 5B) is recognized as the rate-limiting step for PFOA mineralization.^33,46^ Generating the CF_3_(CF_2_)_n_^•^ radical is also important because it undergoes a series of defluorination events via reactions involving ^•^OH (*vide infra*).^47^ Potentials >2 V vs. Ag/AgCl were observed during PFOA oxidation in this study (Figure S5); these potentials are sufficient to oxidize the supporting HCO_3_^–^ electrolyte (Figure 5C).^48^ We hypothesized that HCO_3_^–^ oxidation generates CO_2_, which may carboxylate the CF_3_(CF_2_)_n_CO_2_^•^ radical (retro-PFOA-decarboxylation) according to Le Chatelier’s principle. Accordingly, we performed CCE under ^13^CO_2_ (instead of N_2_) to test this hypothesis. The optimized heterogeneous conditions, which previously degraded PFOA by 84% and afforded a defluorination rate of 91%, led to a PFOA degradation rate of 72% and a free-F^–^ recovery rate of 51.2% in ^13^CO_2_-saturated 0.1 M KHCO_3_. **PFO**-^**13**^ **CO**_**2**_ **H** should be produced via retro-PFOA-decarboxylation if the CF_3_(CF_2_)_n_^•^ radical produced in the initial PFOA decarboxylation step reacts with ^13^CO_2_ (Figure 5D). We used ^13^C NMR spectroscopy to confirm that **PFO**-^**13**^ **CO**_**2**_ **H** is indeed produced. The pre-CCE solution exhibited a ^13^C signal for HCO_3_^–^ at −160 ppm, with no additional ^13^C signal observed after saturating the pre-CCE electrolyte solution with ^13^CO_2_, as expected (Figure 5E,5F). While we did not observe any ^13^C signal for the PFOA backbone owing to the low (2 mM) PFOA concentration in the electrolyte solution, 16 h of CCE led to a new strong ^13^C signal at −164 ppm (Figure 5F) that matched the ^13^C signal for the CO_2_H group in PFOA.^33^ Furthermore, we also noted the PFOA degradation rate across various KHCO_3_ concentrations ([KHCO_3_]), with high levels of PFOA degradation and F^–^ recoveries obtained at [KHCO_3_] < 0.1 M (Figure 5G). In contrast, high [KHCO_3_] values of 0.5 and 1.0 M led to less PFOA degradation, namely ∼70 and 50%, respectively (Figure 5G), which also supports our hypothesis that the CO_2_ generated in the solution can reverse the PFOA decarboxylation process, leading to a lower degree of PFOA degradation. In addition, no free F^–^ was observed at high [KHCO_3_] (Figure 5G), which further indicates that reverse PFOA decarboxylation leads to a smaller amount of CF_3_(CF_2_)_n_^•^ capable of being defluorinated. The ability of bicarbonate to scavenge hydroxyl radicals is another factor that contributes to lower degradation rates at higher bicarbonate concentrations. A higher bicarbonate concentration was previously found to suppress the rate of degradation of nonafluoro-3,6-dioxaheptanoic acid by consuming hydroxyl radicals, which are key radical species generated during the electrochemical oxidation reaction.^49^ Our KHCO_3_-concentration-dependent experiments revealed that the rate of PFOA degradation decreases with increasing bicarbonate concentration (Figure S33), which supports the hypothesis that more hydroxyl radicals are scavenged at higher bicarbonate concentrations, leading to lower rates of PFOA degradation and defluorination.

**Figure 5:**
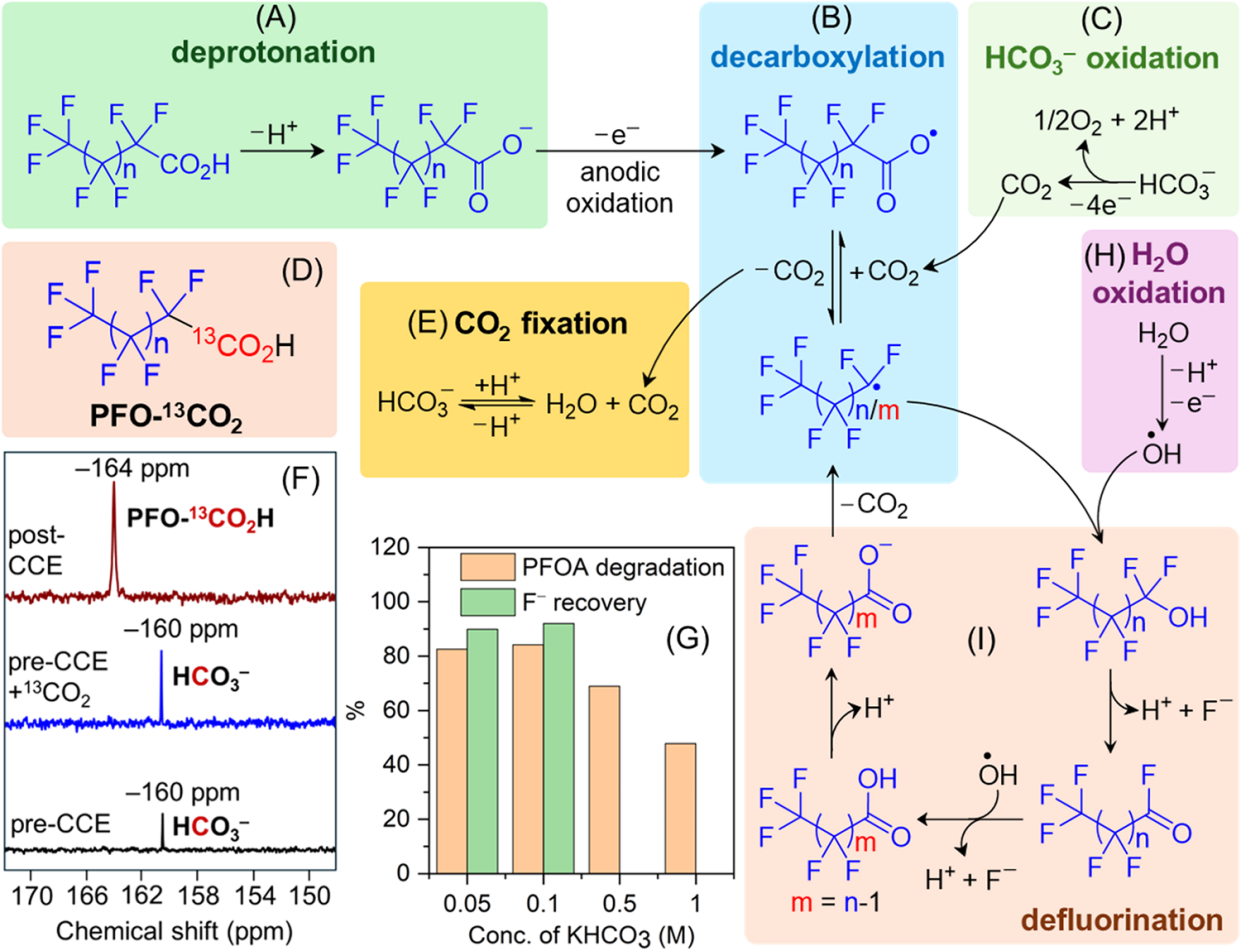
(A) Deprotonation of PFOA. (B) Decarboxylation of PFOA. (C) Electrochemical oxidation of HCO_3_^–^. (D) The structure of isotopically labeled PFOA. (E) Reaction of the CO_2_ produced during the PFOA decarboxylation reaction with H_2_O. (F) ^13^C NMR spectra of the pre-CCE KHCO_3_ electrolyte (black), after saturating with ^13^CO_2_ (blue), and after CCE (maroon) under ^13^CO_2_. (G) PFOA degradation and F^–^ recovery rates obtained after controlled-current electrolysis (CCE) at 5 mA over 18 h at [KHCO_3_] values of 0.05, 0.1, 0.5, and 1.0 M using **[CuT2]** ^**+**^-adsorbed carbon paper electrodes in the presence of 2 mM PFOA. (H) Electrochemical oxidation of water. (I) Defluorination events during PFOA degradation.

The high potential (>2 V vs. Ag/AgCl) observed during PFOA oxidation is sufficient to oxidize the aqueous medium, with ^•^OH radicals expected to be produced (Figure 5H). CF_3_(CF_2_)_n_^•^ reacts with ^•^OH during the electrochemical oxidation of PFOA to produce CF_3_(CF_2_)_n_OH (Figure 5I), which undergoes a first round of defluorination,^44,45^ with CF_3_(CF_2_)_n_(CO)F generated as a consequence, which then becomes involved in another defluorination step to yield CF_3_(CF_2_)_m_CO_2_H (m = n–1) via reaction with another ^•^OH radical. CF_3_(CF_2_)_m_CO_2_H (or the deprotonated form, CF_3_(CF_2_)_m_CO_2_^–^) then becomes involved in another round of decarboxylation and defluorination steps, with multiple rounds affording free F^–^ as the major product and short-chain PFAS by-products, as detected by ESI-MS (Figures S28 and S29). Moreover, we degraded PFOA in the presence of methanol (as a radical scavenger) in the anolyte solution to confirm that the defluorination process is initiated by CF_3_-(CF_2_)_n_^•^ during the electrooxidation of PFOA. The addition of 1.5 M methanol led to declines in the PFOA degradation and defluorination rates (from 84.2 to 64.3%, and 91 to 69.4%, respectively) when the post-CCE solution was analyzed after CCE at 5 mA for 18 h using the heterogenized **[CuT2]** ^**+**^ carbon anode. The electrical energy per order (EE/O) for electrochemical mineralization of PFOA is calculated to evaluate the energy efficiency. The EE/O for our **[CuT2]** ^**+**^ deposited carbon electrode system is calculated to be 86.2 kWhm^–3^, which is comparatively lower than the other reported electrochemical oxidation,^47,50^ sonochemical,^51^ photochemical,^52^ and UV/KI systems^53^ (Table S2).

We explored the effects of other supporting electrolytes during aqueous PFOA degradation promoted by the heterogenized Cu­(I) catalyst by performing CCE at 5 mA over 24 h in the presence of 0.1 M NaClO_4_, LiClO_4_, Na_2_SO_4_, or KCl. All supporting electrolytes exhibited PFOA degradation rates in excess of 90% under identical heterogeneous conditions, but with poor F^–^ recoveries (∼36% for NaClO_4_/LiClO_4_; 20–28% for Na_2_SO_4_/KCl), unlike the high F^–^ yield obtained with 0.1 M KHCO_3_. To ensure the meaningful comparison between different supporting electrolytes, all the experiments were conducted under comparable pH conditions of 9.1, consistent with the HCO_3_^–^ electrolyte system and no significant differences were observed in degradation and fluoride recovery. Although the reasons for the poor activities of these electrolytes are not well understood, these results suggest that the HCO_3_^–^-containing supporting electrolyte influences PFOA decarboxylation and defluorination uniquely in aqueous media.

### Degrading Other PFAS Substrates

We investigated the catalytic activity of **[CuT2]** ^**+**^-drop-cast carbon anodes for disintegrating other PFAS substrates (Figure 6A–C) via CCE at 5 mA for 18 h in N_2_-saturated 0.1 M KHCO_3_ containing 2 mM PFAS (Figures S34–S53). We examined three other PFCAs: perfluoropropionic acid (PFPA), perfluorobutanoic acid (PFBA), and perfluoroheptanoic acid (PFHA), and five PFDCAs: difluoropropanedioic acid (DFPDA), tetrafluorobutanedioic acid (TFBDA), hexafluoropentanedioic acid (HFPDA), octafluorohexanedioic acid (OFHDA), and hexadecafluorodecanedioic acid (HDFDA). We also included two other FTCAs, namely pentafluoropentanoic acid (PFPC2A) and heptafluorohexanoic acid (HFHC2A). The PFAS degradation and F^–^ recovery rates obtained using these substrates under identical CCE conditions are shown in Figure 6D–F (also see Table S3). Overall, these data reveal that the rate of degradation declines from 87 to 34% as the number of carbons in the chain is decreased from seven to three, which suggests that the rates of PFAS degradation and defluorination depend on the length of the fluoroalkyl chain, consistent with theoretical predictions.^54^ Furthermore, shorter-chain PFASs exhibited comparatively lower rates of free F^–^ formation, in good agreement with previously reported data.^44^

**Figure 6:**
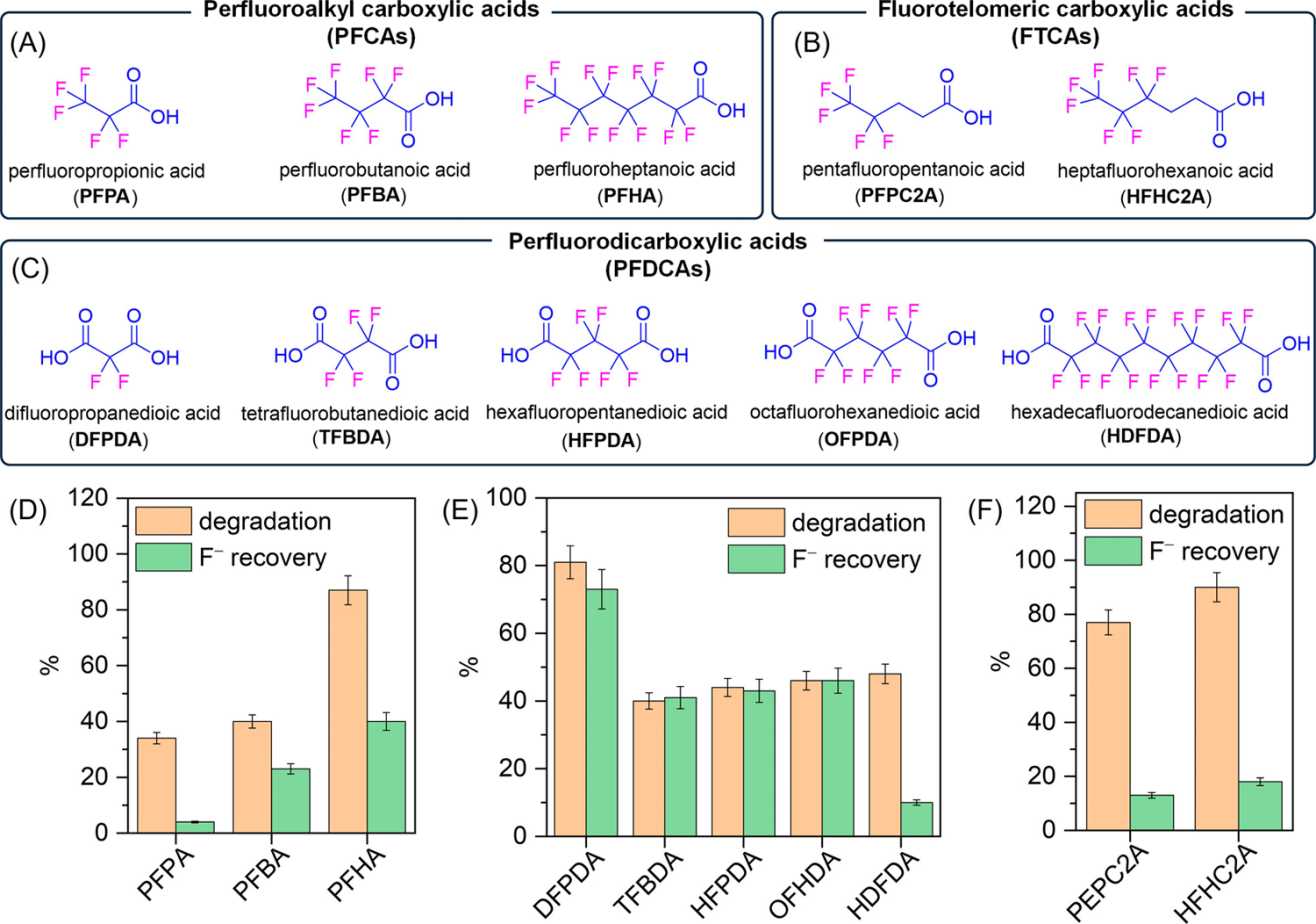
(A) Perfluoroalkyl carboxylic acids (PFCAs), (B) fluorotelomeric carboxylic acids (FTCAs), and (C) perfluorodicarboxylic acids (PFDCAs) investigated in this study. Degradation and F^–^ recovery rates obtained over the **[CuT2]** ^**+**^-adsorbed carbon electrode after controlled-current electrolysis at 5 mA for 18 h in 0.1 M KHCO_3_ containing (D) PFCAs, (E) PFDCAs, and (F) FTCAs.

### Fluorinating Alkyl/Aryl Sulfonyl Chlorides

The free F^–^ ions produced during the anodic oxidation of PFOA were repurposed as nucleophiles for the synthesis of alkyl/aryl sulfonyl fluorides from their corresponding chloride derivatives (Figure 7A). Fluorination reactions were carried out *in-situ* without extracting F^–^ from the PFOA-degraded post-CCE solutions. A higher concentration of F^–^ was obtained by dissolving PFOA in aqueous 0.1 M KHCO_3_ electrolyte to a concentration of 36 mM by adding 10% (v/v) MeCN, with CCE performed at 5 mA and terminated after 18 h. MeSO_2_Cl (35 mM) was then added to the post-CCE anolyte and the mixture was stirred at room temperature without any applied current. Samples collected for ^19^F NMR spectroscopy exhibited a signal at 59.61 ppm that evolved with time (Figure 7B). This ^19^F NMR signal matched that of MeSO_2_F, confirming that the desired fluorination event had occurred.^39^ The highest yield of MeSO_2_F (94.3%) was recorded after 6 h of stirring using tetrabutylammonium hexafluorophosphate (TBAPF_6_) as the internal standard (Figure 7C). Furthermore, Cl­(CH_2_)_2_SO_2_F and BzSO_2_F were produced under identical conditions from their corresponding chloride derivatives in yields of 82.9% and 17.1%, respectively (Figures S54 and S55). Remarkably, these results indicate that alkyl/aryl sulfonyl chlorides can be fluorinated *in situ* using PFAS-derived fluoride in the absence of any external energy or chemical inputs, such as current, heat, or pressure. Notably, the ^19^F NMR signals for PFOA were also observed to decrease when the solution was stirred during the *in-situ* fluorination process (Figure 7B). As no current was applied when the post-CCE anolyte was stirred during fluorination, we propose that the decrease in the ^19^F NMR PFOA signal is possibly ascribable to ^•^OH formed during the oxidation of H_2_O, as discussed earlier (Figure 5I); these radicals continue to defluorinate PFOA molecules. In addition, the signal for free F^–^ also slowly disappeared as the *in-situ* fluorination process progressed, indicative of the consumption of free F^–^ in the anolyte solution. However, there is no direct correlation between F^–^ consumption and the formation of fluorinated products as F^–^ was also observed in the counter electrochemical compartment, indicative of F^–^ migration through the membrane while the solution was stirred. A controlled experiment without stirring was performed to confirm that F^–^ does not pass through the membrane.

**Figure 7:**
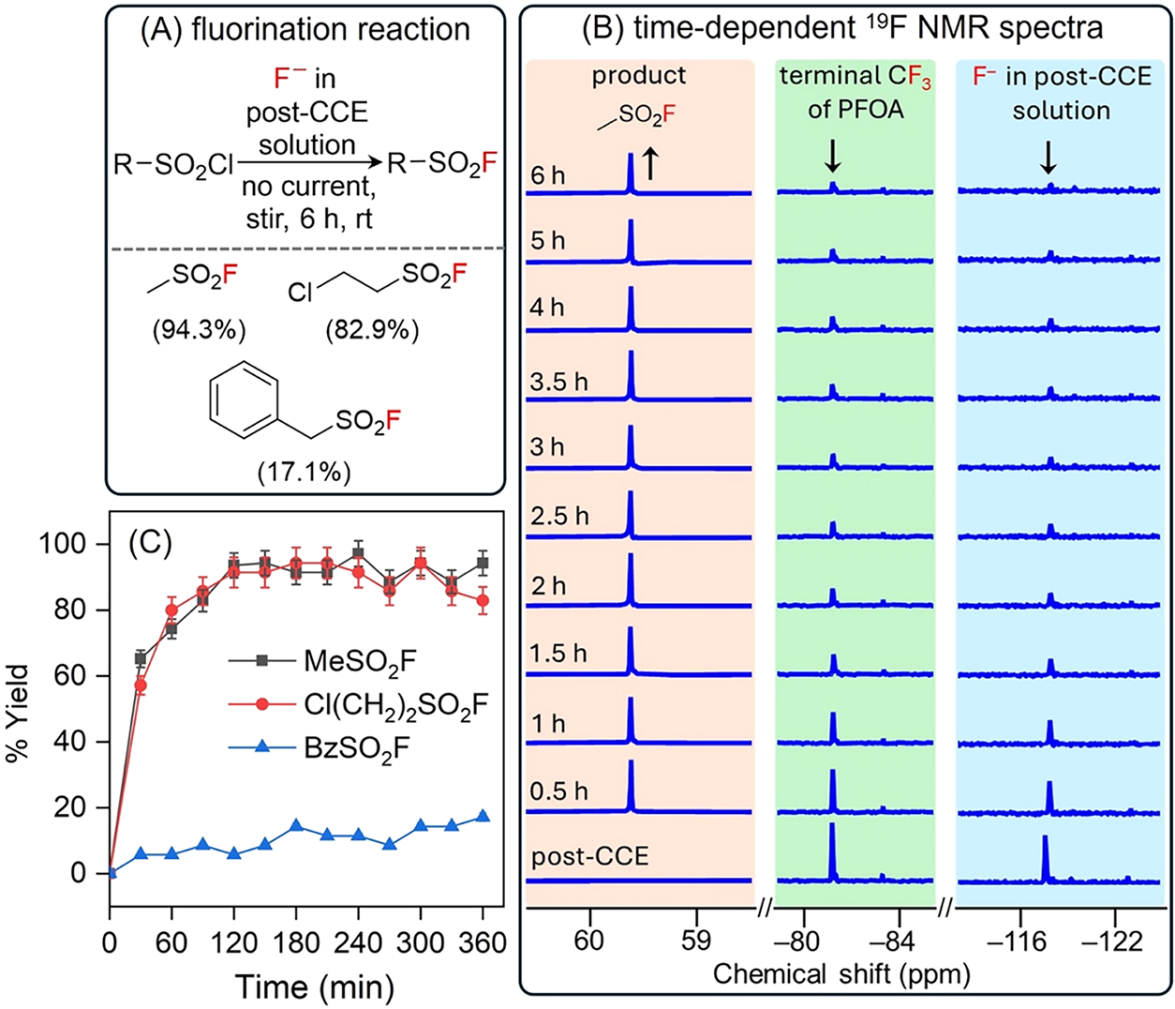
(A) General reaction scheme for the fluorination of alkyl/aryl (R) sulfonyl chlorides using the free F^–^ present in the post-controlled-current-electrolysis (CCE) solution after completion of the PFOA oxidation reaction. (B) Time-dependent ^19^F NMR spectra recorded for the stirred post-CCE solution after adding MeSO_2_Cl in the absence of an applied current. (C) Yields of the MeSO_2_F, Cl­(CH_2_)_2_SO_2_F, and BzSO_2_F products from the reactions of their corresponding chloride derivatives with the free F^–^ in the post-CCE solution.

## Conclusions

Herein, we developed a heterogenized-copper­(I)-catalyst-adsorbed carbon anode for electrochemical PFAS mineralization in aqueous media. We previously explored this copper­(I) catalyst in its molecular form for the degradation of PFOA in a nonaqueous electrolyte under reductive conditions. Interestingly, the same copper­(I) catalyst exhibited an impressive ability to promote the oxidative degradation of PFOA, with an 84% degradation rate observed in aqueous media when immobilized on carbon paper. This is a rare example of a molecular catalyst capable of both oxidative and reductive PFOA degradation under heterogeneous and homogeneous conditions. Furthermore, this study also elucidated the role played by the HCO_3_^–^ electrolyte in the decarboxylation of PFOA, the rate-limiting step of the PFOA-degradation process, as revealed by CCE experiments performed in ^13^CO_2_-saturated aqueous KHCO_3_ electrolyte. In addition, we present degradation and F^–^ recovery rates for the heterogenized copper­(I) catalyst toward the degradation of diverse groups of PFAS under identical conditions. Finally, we demonstrated that the produced F^–^ can be upcycled for the *in-situ* formation of fluorinated products of medicinal importance, particularly MeSO_2_F, which is used to treat Alzheimer’s disease. We believe that this study not only provides examples of both molecular and heterogeneous catalysts, but is expected to be of interest for electrochemically activating strong C–F bonds and the use of F^–^ for the production of fluorinated medicinal agents, which remains a medicinal chemistry challenge.

## Experimental Section

### Materials

All PFAS substrates discussed in this study, KHCO_3_ (99.7%), Nafion, potassium hexafluorophosphate (KPF_6_), and TBAPF_6_ were purchased from Fisher Scientific or Sigma-Aldrich and used as received. Carbon papers (GDS 2050) for CCE were purchased from the Fuel Cell Store. The synthesis procedure and characterization data of **[CuT2]** ^**+**^ are reported in our previous publication.^33^ We used deionized water (18 MΩ cm at 22 °C) from a Millipore water purification system in all experiments.

### Characterization

^1^H and ^19^F NMR were recorded by a Bruker AV400 MHz NMR instrument. Gaseous products were analyzed by GC (MG #5, SRI Instruments) equipped with a flame ionization detector and a thermal conductivity detector. The amount of CO_2_ generated from the PFOA oxidation was determined by subtracting the CO_2_ generated during oxidation of only bicarbonate (without PFOA) from the CO_2_ generated during oxidation of PFOA in bicarbonate. IC analysis was carried out on Metrohm equipped with Metrosep A Supp 5–150/4.0 column attached with a Metrosep A Supp 5 Guard/4.0. 0.1 M H_2_SO_4_ was passed as suppressor regenerant with the standard aqueous eluent supplied from the Metrohm. ESI-MS analysis was carried out on a Finnigan Fourier Transform mass spectrometer (LTQ FT-MS) from Thermo Scientific. All electrochemical measurements were carried out on a Bio-Logic VSP potentiostat.

### Sample Preparation for NMR and IC

The pre- and post-electrolysis solutions were analyzed by ^19^F NMR by mixing 400 μL of anolyte solution, 100 μL of D_2_O, and 25 μL of aqueous 0.1 M KPF_6_ (internal standard). The time-dependent ^19^F NMR spectra for the post-CCE solutions were recorded upon adding MeSO_2_Cl, Cl­(CH_2_)_2_SO_2_Cl, or BzSO_2_Cl by mixing 300 μL of post-CCE anolyte solution, 100 μL of CD_3_CN, and 50 μL of 0.1 M TBAPF_6_ internal standard in acetonitrile. The product yield of the fluorinated compounds is calculated by using crude post-electrolysis solutions upon integrating ^19^F NMR signals in the presence of TBAPF_6_ as an internal standard. To measure the concentration of F^–^ through IC, the post-CCE solution was diluted by mixing 0.5 mL of the post-CCE solution in 0.5 mL of water (total volume = 1 mL).

### Preparation of Electrodes

The carbon paper working electrodes for CCE experiments were prepared by dissolving 1.5 μmol of **[CuT2]** ^**+**^ in 100 μL of a Nafion solution (95% propanol, and 5% Nafion dispersion, volume percentage). The mixture was sonicated for 15 min to achieve good dispersion, which was subsequently deposited on the bottom 1 cm^2^ of GDS 2050 carbon paper (3 cm × 0.5 cm, Fuel Cell Store). The electrode was then dried overnight and used as the working electrode.

### Controlled-Current Electrolysis (CCE)

A custom designed gas tight H-cell, with Selenion DSV anion-exchange membrane between the working and counter cell compartments, was used for all electrolysis experiments. The working cell contained 5 mL of 0.1 M supporting electrolyte containing aqueous solution with 2 mM of PFOA unless otherwise noted. All the electrochemical reactions were performed at room temperature and atmospheric pressure. The setup featured a **[CuT2]** ^**+**^-immobilized GDS 2050 carbon paper working electrode, Pt-plate counter electrode, and Ag/AgCl (4 M KCl) reference electrode for CCE experiments. The working cell was sealed using an airtight custom Teflon cap equipped with tubing to channel any gaseous products in the headspace to the gas chromatograph for analysis. To ensure all gaseous products were accurately conveyed to the gas chromatography system, each assembly of the cell underwent leak tests. The gaseous products in the anodic chamber were injected into the sample loop of the gas chromatograph with the flow of N_2_ gas. N_2_ gas was used as a carrier gas within the system.

### Inductively Coupled Plasma Mass Spectrometry (ICP-MS)

The copper present on the **[CuT2]** ^**+**^ immobilized carbon paper working electrode before and after the CCE was analyzed by using ICP-MS and obtained to be 0.173 and 0.165 μmol, respectively. The concentration of copper was calculated based on the calibration curve plotted (see Supporting Information). The electrode surface was thoroughly rinsed with acetonitrile, and the catalyst was digested in aqua regia. It was diluted using 2% nitric acid to the required volume to bring it within the calibrated range.

### Calculations of Overall %fluoride Recovery

The overall %fluoride recovery rate of the pre and post-solutions are calculated based on eq 1.

1 $$Overall\,\;\%fluoride\,\;recovery=\frac{\left[F^{-}\right]}{{\left[PFOA\right]}_{de\mathrm{grad}ed}\times N_{C-F}}\times 100$$

where, [F^–^] is the measured fluoride ion concentration of the solutions. [PFOA]_degraded_ is the concentration of PFOA degraded during the electrolysis and N_C–F_ is the total number of C–F bonds in the parent PFOA molecule.

### Calculations for Coulombic Efficiencies

The coulombic efficiency (CE) for fluoride recovery is calculated with the following eq 2.

2 $$\mathrm{CE}=\frac{FVe\left[F^{-}\right]}{At}$$

where, *V* = volume of anolyte (0.005 L)

e = number of electrons needed to produce one fluoride ion

[F^–^] = concentration of fluoride ions (mol/L)

A = applied current (ampere)

*t* = time of electrolysis (second)

### Electrical Energy Per Order (EE/O) Calculation

The electrical energy per order (EE/O) is defined as the amount of energy (kWh m^–3^) required to reduce the pollutant by one order of magnitude. The energy consumption for this system was calculated to be 86.2 Wh L^–1^ or kWh m^–3^. The electrical energy per order (EE/O) is calculated using the following eq 3.

3 $$\mathrm{Electrical}\,\;\mathrm{energy}\,\;\mathrm{per}\,\;\mathrm{order}\left(\mathrm{EE}/O\right)=\frac{U\times I\times t}{\log\left(\frac{C_{i}}{C_{f}}\right)\times V}$$

where, *U* = average cell potential (V)

*I* = amount of applied current (A)

*t* = time of electrolysis (h)

*C_i_* = initial concentration of PFOA (mM)

*C_f_* = final concentration of PFOA (mM)

*V* = volume of electrolytic solution (L)

## Supplementary Material



## Notes

Electrochemical and spectroscopy data (figures and tables) (PDF)
