# Supplementary material for: Heterogeneous PFAS Defluorination and Fluoride Upcycling into Medicinal Agents in Aqueous Electrolytes
Source: ACS Electrochem. 2026 May 1;2(6):1422–32. doi: 10.1021/acselectrochem.6c00070 (PMC13244370; doi:10.1021/acselectrochem.6c00070)
Supplement: Supplementary file 1 [file ec6c00070_si_001.pdf]

Supporting Information

Heterogeneous PFAS Defluorination and Fluoride Upcycling into Medicinal Agents in Aqueous Electrolytes

Pramod Tiwari,[†] Nabin Pandey,[†] Soumalya Sinha,[†,*] and Jianbing "Jimmy" Jiang[†,*]

[†]Department of Chemistry, University of Cincinnati, Cincinnati, OH 45221, United States

*Corresponding authors: Email: sinhaso@uc.edu and jianbing.jiang@uc.edu

Table of Contents **Page**

Table of Contents	Page
Figure S1. SEM image of bare carbon electrode	S5
Figure S2. EDX data recorded for the **[CuT2]**$^{+}$ deposited carbon electrode	S5
Figure S3. EDX data recorded for the bare carbon paper electrode	S6
Figure S4. Gas chromatography traces for CO_2 evolution	S6
Figure S5. CCE trace for PFOA oxidation at 5 mA for 18 h	S7
Figure S6. ^{19}F NMR spectrum recorded for potassium fluoride	S7
Figure S7. ^{19}F NMR spectrum of PFOA before and after CCE at 5 mA	S8
Figure S8. IC obtained for post-electrolysis solution at 5 mA for 18 h	S9
Figure S9. IC calibration curve	S9
Figure S10. CCE experiment to check the self degradation of PFOA	S10
Figure S11. ^{19}F NMR spectrum recorded for post-CCE without application of current	S11
Figure S12. IC obtained for post-CCE without application of current	S12
Figure S13. ^{19}F NMR spectrum recorded for post-CCE without catalyst	S13
Figure S14. ^{19}F NMR spectrum recorded for post-CCE using CuO deposited electrode	S14
Figure S15. IC obtained for post-CCE using CuO deposited electrode	S15
Figure S16. ^{19}F NMR spectrum recorded for post-CCE without PFOA	S16
Figure S17. IC obtained for post-CCE without PFOA	S17
Figure S18. Potential vs. time profile recorded for **[CuT2]**$^{+}$ and Nafion	S18
Figure S19. CCE trace of PFOA oxidation at 5, 7.5, and 10 mA for 8 h	S19
Figure S20. ^{19}F NMR spectrum of PFOA oxidation at 5, 7.5, and 10 mA for 8 h	S20
Figure S21. IC data obtained for of PFOA oxidation at 5, 7.5, and 10 mA for 8 h	S21
Figure S22. ^{19}F NMR of post-CCE solution of 0.5 mM PFOA at 5 mA	S22
Figure S23. IC trace for post electrolysis solution of 0.5 mM PFOA	S23
Figure S24. ^{19}F NMR of post-CCE solution of 1 mM PFOA at 5 mA	S24
Figure S25. IC trace for post electrolysis solution of 1 mM PFOA	S25
Figure S26. ^{19}F NMR post-CCE solution of 1.5 mM PFOA at 5 mA	S26

Figure S27. IC trace for post electrolysis solution of 1.5 mM PFOA S27

Figure S28. ESI-MS data collected for the pre-CCE solution of PFOA S28

Figure S29. ESI-MS data collected for the post-CCE solution of PFOA S29

Figure S30. XPS of bare carbon paper electrode S30

Figure S31. XPS of rinsed **[CuT2]**$^+$ carbon paper electrode S31

Figure S32. ICP-MS calibration curve for the determination Cu concentration S32

Figure S33. PFOA degradation at different $KHCO_3$ concentrations S33

Figure S34. ^{19}F NMR spectrum pre-CCE solution of PFPA S34

Figure S35. ^{19}F NMR spectrum post-CCE solution of PFPA at 5 mA S35

Figure S36. ^{19}F NMR spectrum pre-CCE solution of PFBA S36

Figure S37. ^{19}F NMR spectrum post-CCE solution of PFBA at 5 mA S37

Figure S38. ^{19}F NMR spectrum pre-CCE solution of PFHA S38

Figure S39. ^{19}F NMR spectrum post-CCE solution of PFHA at 5 mA S39

Figure S40. ^{19}F NMR spectrum pre-CCE solution of DFPDA S40

Figure S41. ^{19}F NMR spectrum post-CCE solution of DFPDA at 5 mA S41

Figure S42. ^{19}F NMR spectrum pre-CCE solution of TFBDA S42

Figure S43. ^{19}F NMR spectrum post-CCE solution of TFBDA at 5 mA S43

Figure S44. ^{19}F NMR spectrum pre-CCE solution of HFPDA S44

Figure S45. ^{19}F NMR spectrum post-CCE solution of HFPDA at 5 mA S45

Figure S46. ^{19}F NMR spectrum pre-CCE solution of OFHDA S46

Figure S47. ^{19}F NMR spectrum post-CCE solution of OFHDA at 5 mA S47

Figure S48. ^{19}F NMR spectrum pre-CCE solution of HDFDA S48

Figure S49. ^{19}F NMR spectrum post-CCE solution of HDFDA at 5 mA S49

Figure S50. ^{19}F NMR spectrum pre-CCE solution of PEPC2A S50

Figure S51. ^{19}F NMR spectrum post-CCE solution of PEPC2A at 5 mA S51

Figure S52. ^{19}F NMR spectrum pre-CCE solution of HFHC2A S52

Figure S53. ^{19}F NMR spectrum post-CCE solution of HFHC2A at 5 mA S53

Figure S54. Time-dependent ^{19}F NMR after adding $Cl(CH_2)_2SO_2Cl$ S54

Figure S55. Time-dependent ^{19}F NMR after adding $BzSO_2Cl$ S55

Table S1. Atomic percentage derived from the XPS survey scans. S56

Table S2. Energy consumption of PFOA degradation using different reported methods S56

Table S3. %PFOA degradation and %defluorination for different PFAS substrates S57

References S58

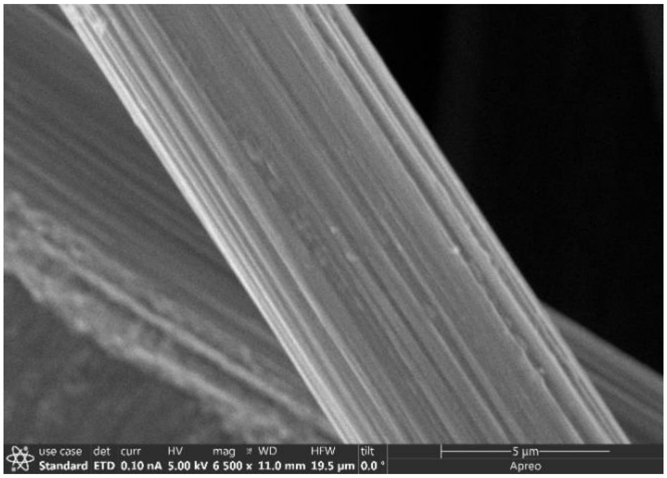

Figure S1. Scanning electron microscope image of bare carbon paper electrode.

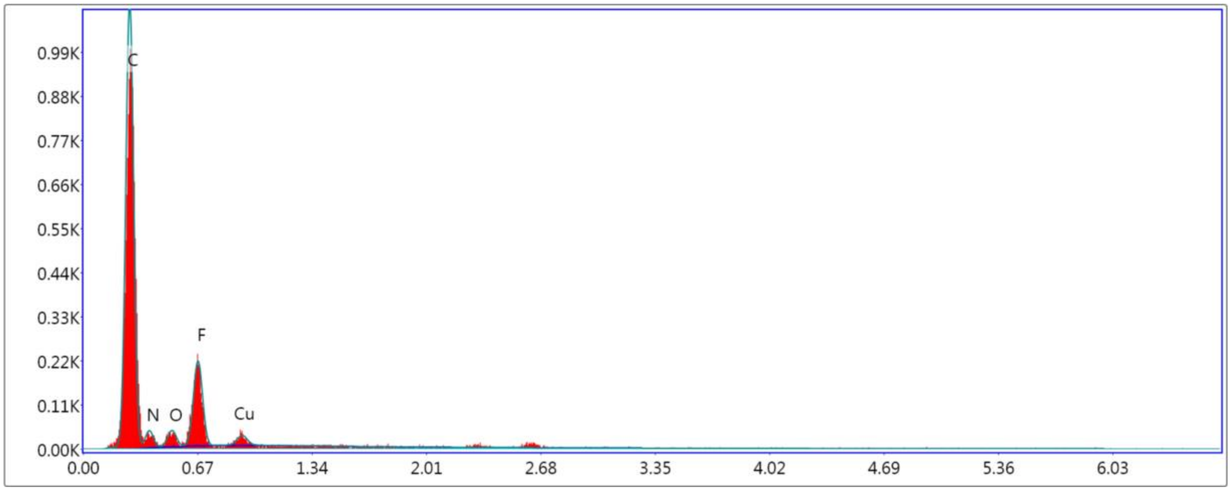

Figure S2. Energy dispersive X-ray spectroscopy data recorded for the **[CuT2]**$^{+}$ deposited carbon paper electrode (GDS 2050) before the controlled current electrolysis. 2.13 weight% and 0.45 atomic% of copper was detected.

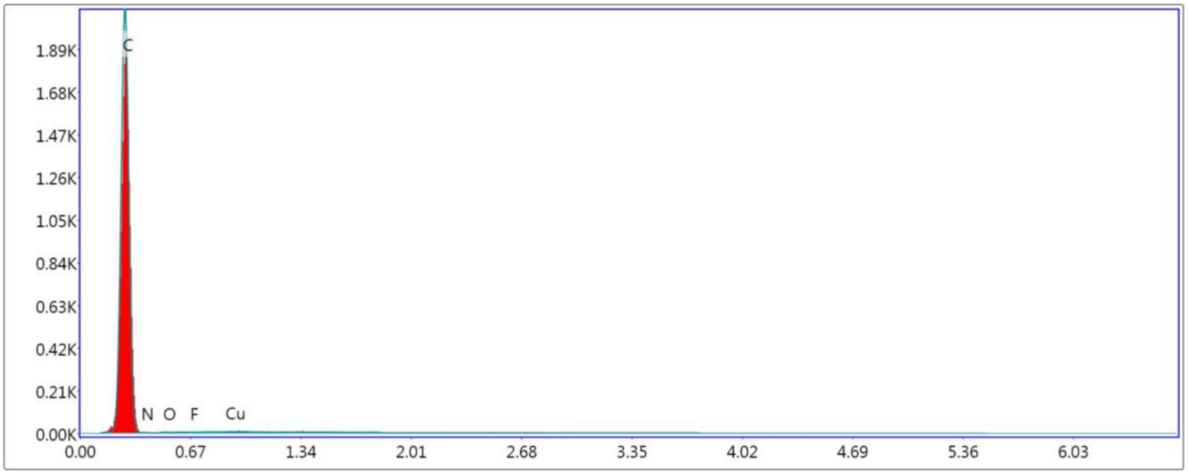

Figure S3. Energy dispersive X-ray spectroscopy data recorded for the bare carbon paper electrode (GDS 2050).

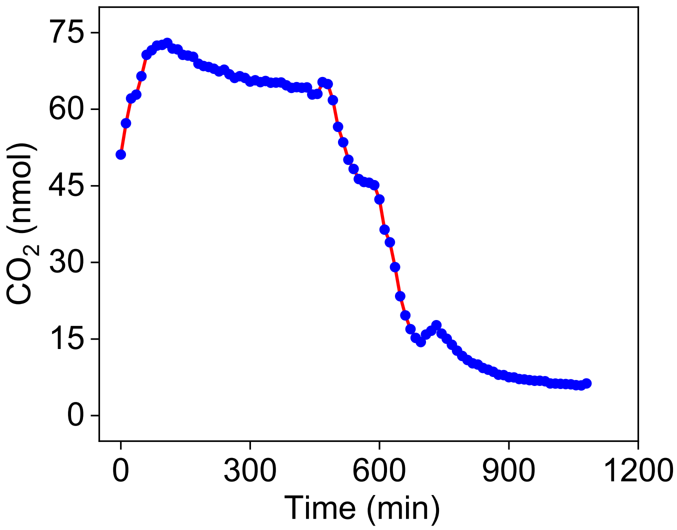

Figure S4. The amount of CO_2 detected by gas chromatography during the controlled-current electrolysis at 5 mA for 18 h using heterogenized **[CuT2]**$^+$ catalyst. The amount of CO_2 generated from the PFOA oxidation was determined by subtracting the CO_2 generated during oxidation of only bicarbonate (without PFOA) from the CO_2 generated during oxidation of PFOA in bicarbonate.

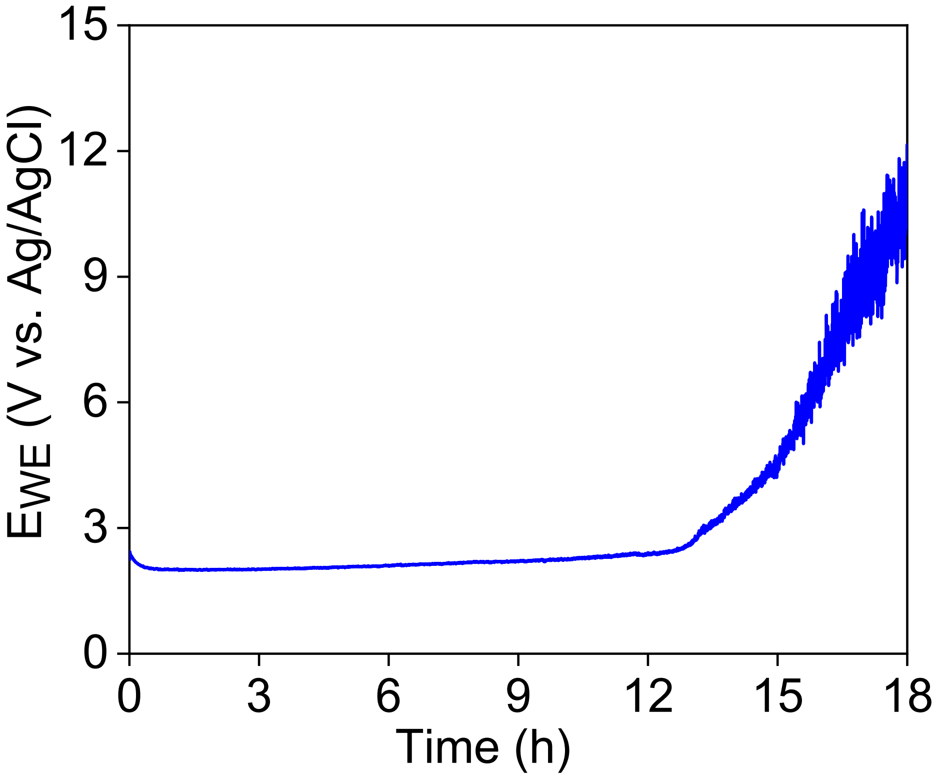

Figure S5. Working electrode potential vs. time profile recorded for PFOA oxidation during controlled-current electrolysis at 5 mA for 18 h using heterogenized **[CuT2]**$^{+}$ catalyst.

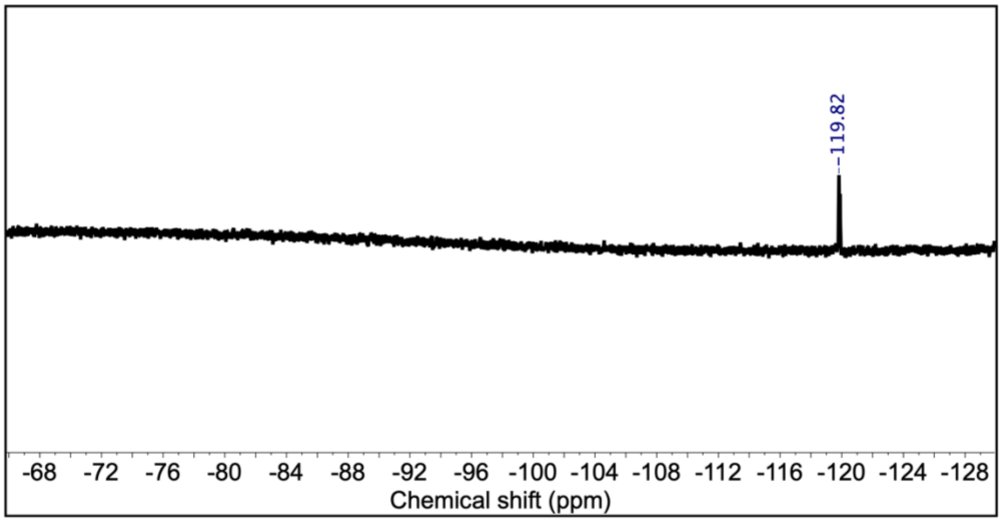

Figure S6. ^{19}F NMR spectrum recorded for a standard sample of potassium fluoride (KF) dissolved in 0.1 M $KHCO_3$ aqueous electrolyte with D_2O.

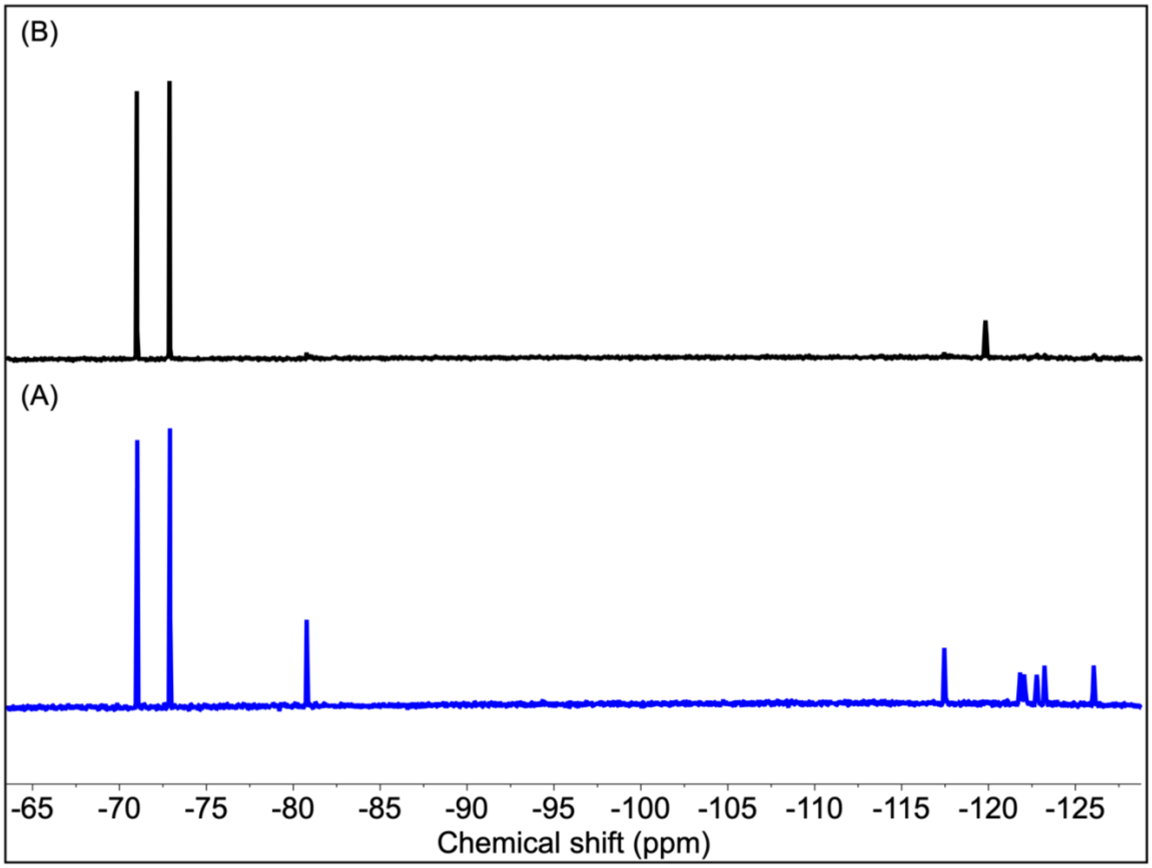

Figure S7. ^{19}F NMR spectrum recorded for 2 mM PFOA in 0.1 M $KHCO_3$. (A) before controlled current electrolysis (CCE) and, (B) after CCE at 5 mA for 18 h. The peaks at –71.02 and –72.91 originate from the internal standard, KPF_6.

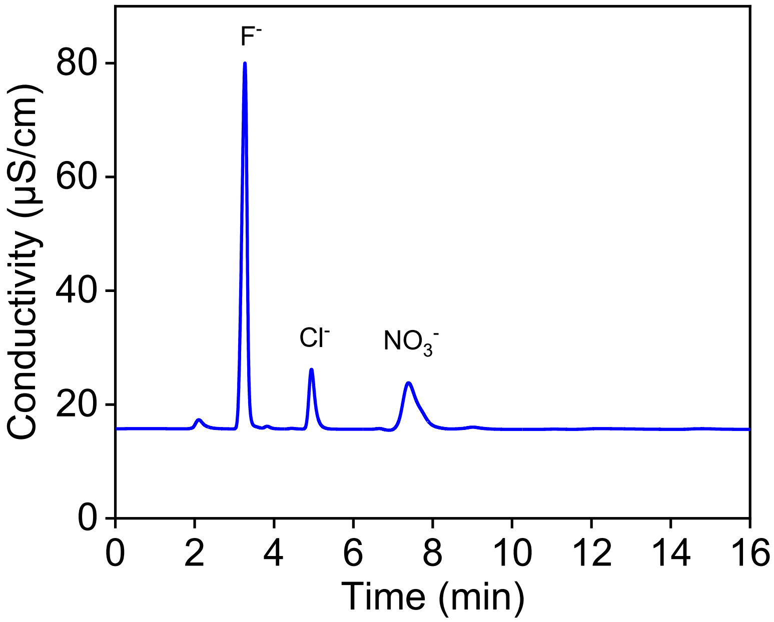

Figure S8. Ion chromatogram collected from post-controlled current electrolysis (CCE) solution after running CCE at 5 mA using heterogenized **[CuT2]**$^+$ catalyst for PFOA degradation in the 0.1 M $KHCO_3$ solution for 18 h. Trace amount of chloride and nitrate ions were identified as common contaminants present in the glassware.

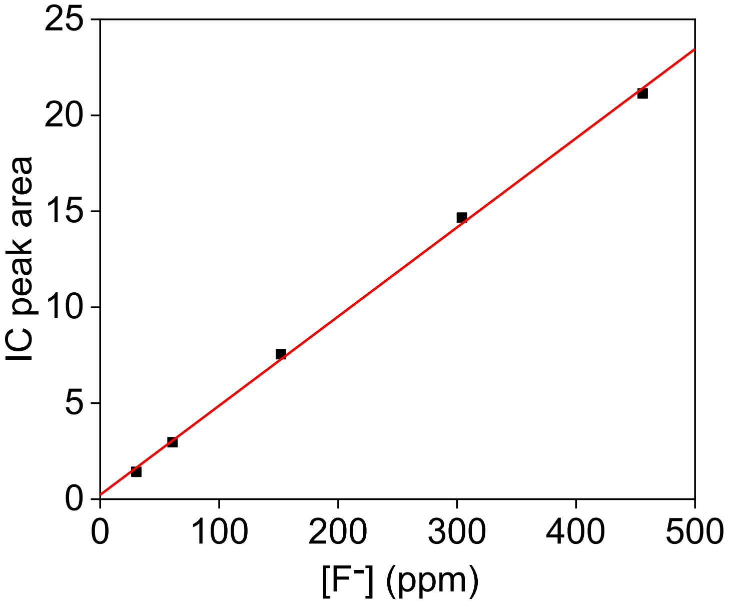

Figure S9. Ion chromatography (IC) calibration curve using standard solutions of potassium fluoride (KF) in deionized water.

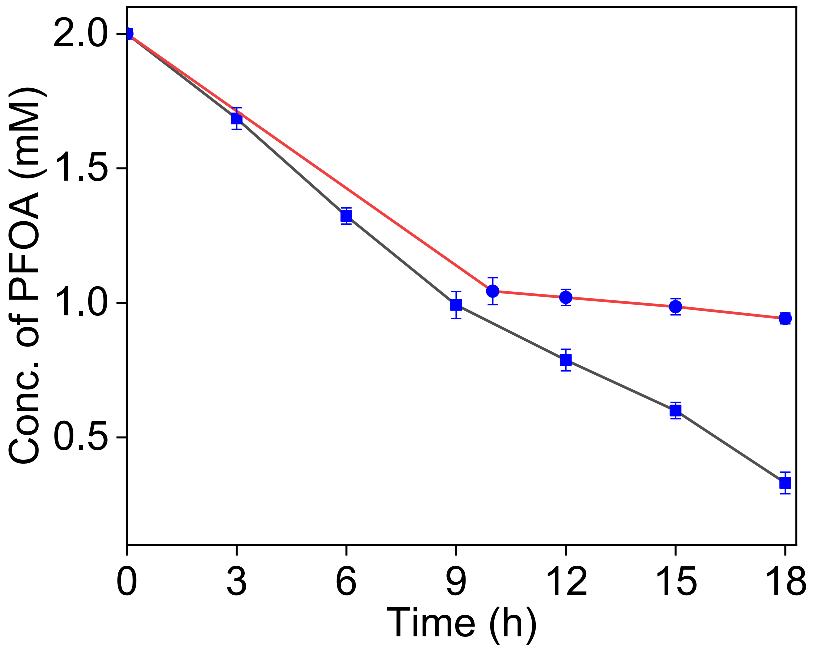

Figure S10. Calculated PFOA concentrations in the electrolytic solution over 18 hours under the electrolysis conditions (black) and PFOA concentration during controlled current electrolysis experiment under identical electrochemical conditions for 10 h, and it was stirred for another 8 h without applying any currents (red).

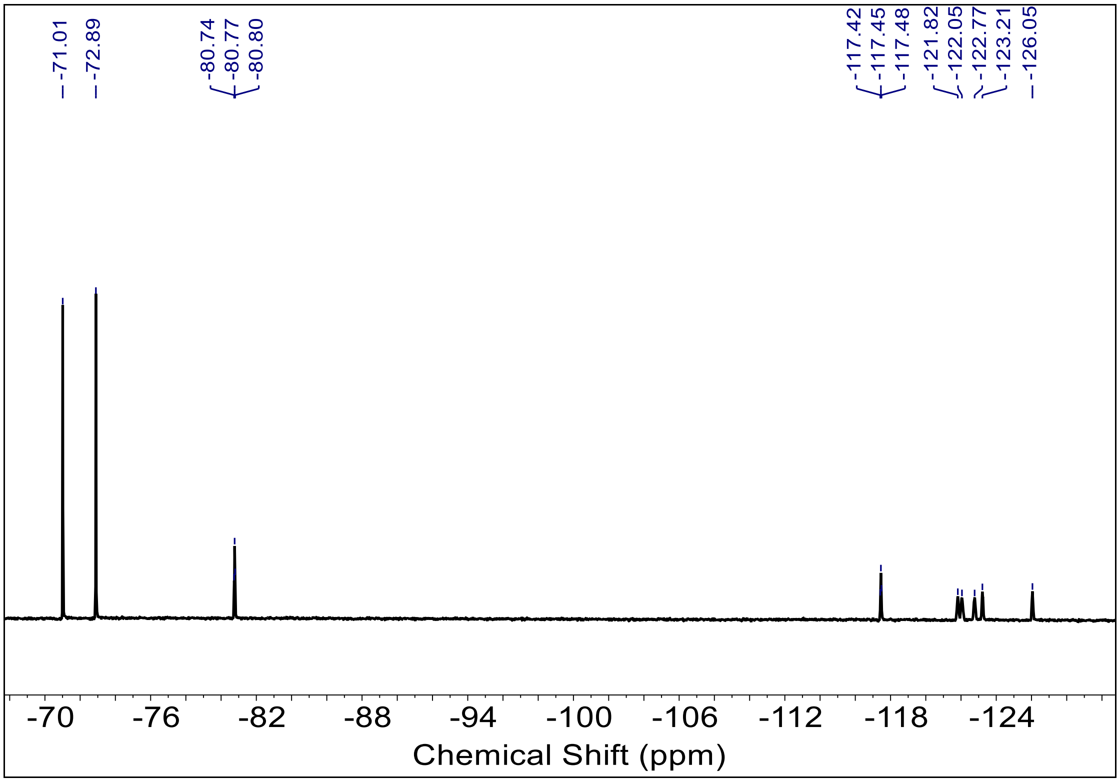

Figure S11. ^{19}F NMR spectrum recorded for the control experiment involving PFOA oxidation in 0.1M $KHCO_3$ at 5 mA for 18 h without application of electric current. The peaks at –71.01 and –72.89 ppm originate from the internal standard, KPF_6.

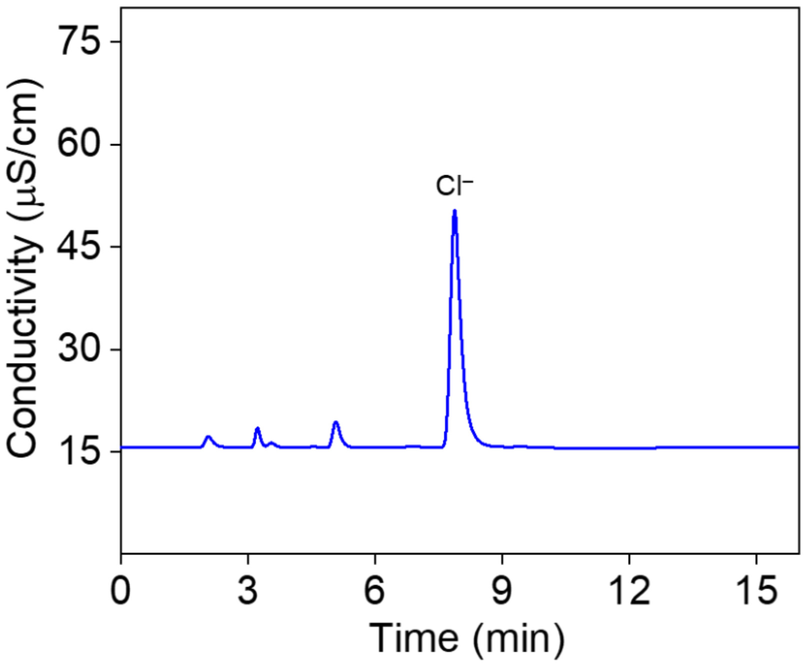

Figure S12. Ion chromatogram collected from post-anolyte solution after 5 mA using heterogenized **[CuT2]**$^{+}$ catalyst for control experiment involving PFOA oxidation in 0.1 M $KHCO_3$ at 5mA for 18 h, but in the absence of applied current.

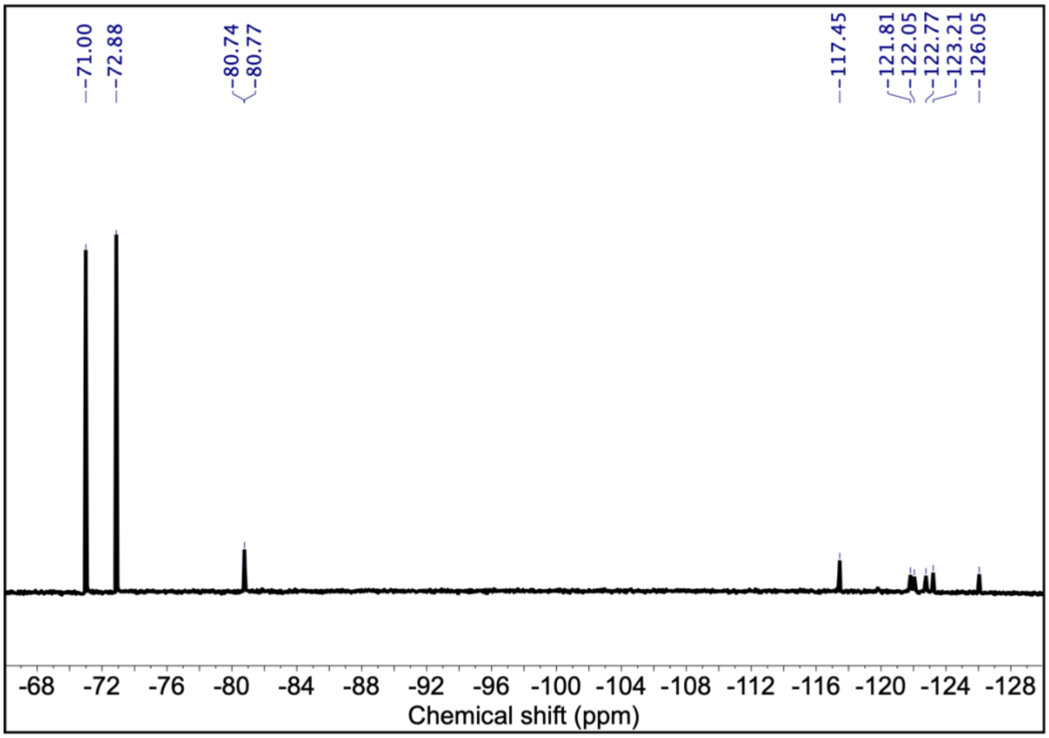

Figure S13. ^{19}F NMR spectrum recorded for post-electrolysis solution of PFOA oxidation at 5 mA for 18 h without drop casting **[CuT2]**$^{+}$ complex. The peaks at –71.00 and –72.88 originate from the internal standard, KPF_6.

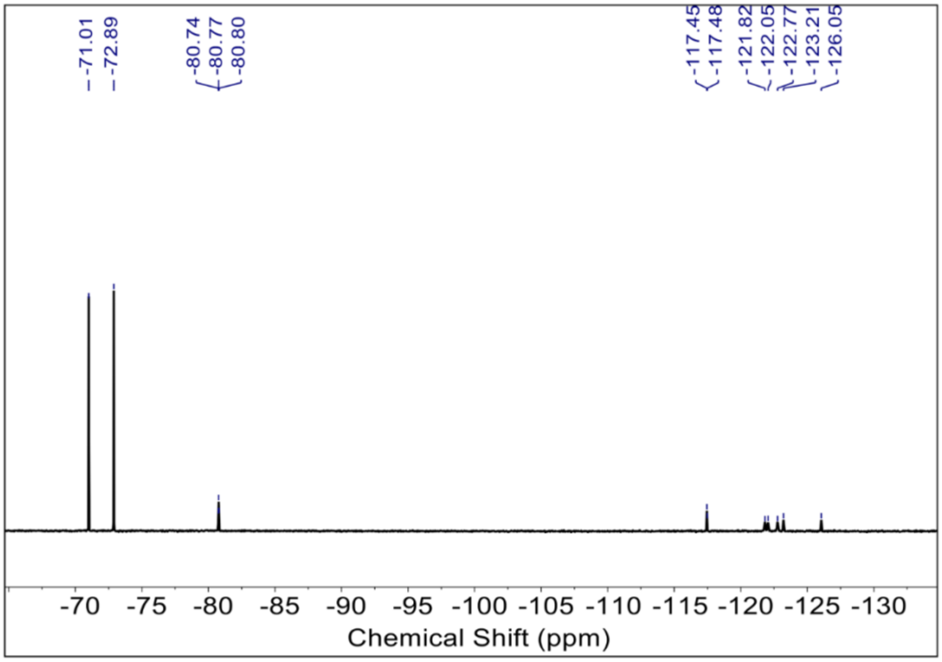

Figure S14. ^{19}F NMR spectrum recorded for the control experiment using a CuO-deposited electrode replacing **[CuT2]**$^{+}$ catalyst deposited electrode for PFOA oxidation. The peaks at –71.01 and –72.89 ppm originate from the internal standard, KPF_6.

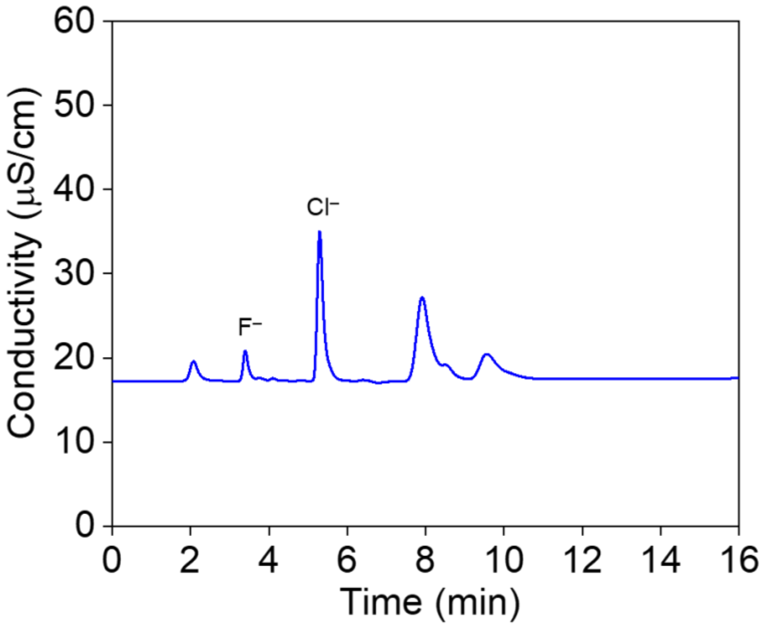

Figure S15. Ion chromatogram collected from post-anolyte solution using a CuO-deposited electrode replacing **[CuT2]**$^+$ catalyst deposited electrode for PFOA oxidation.

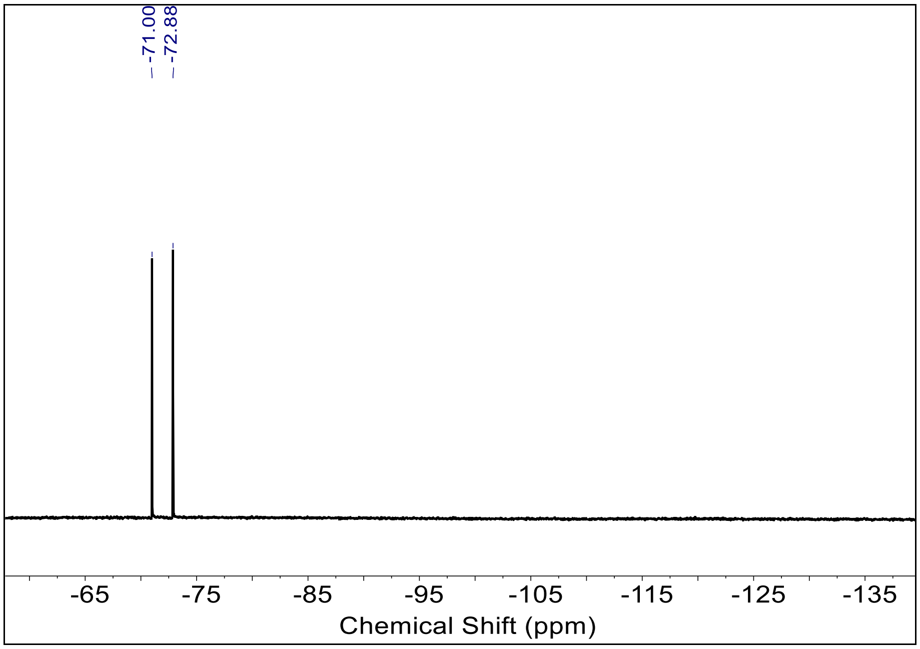

Figure S16. ^{19}F NMR spectrum recorded for control experiment with the heterogenized **[CuT2]**$^{+}$ catalyst deposited electrode but without PFOA in the aqueous electrolyte.

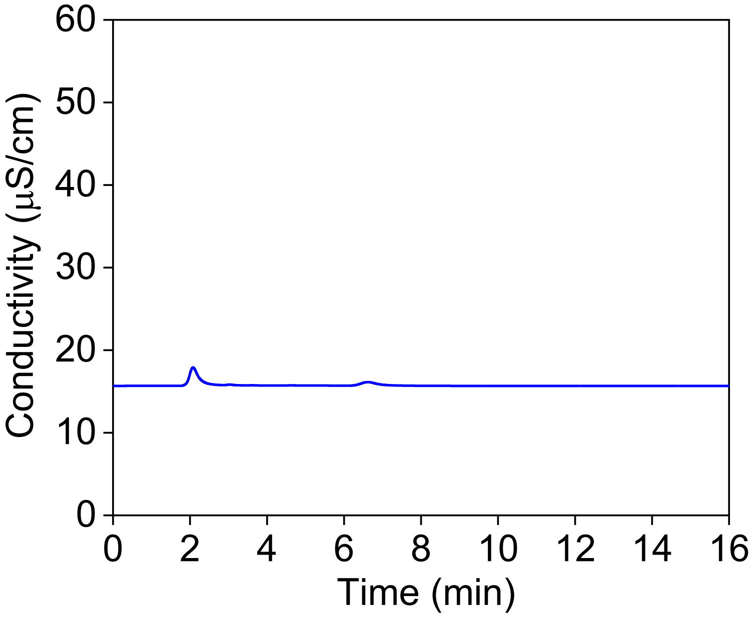

Figure S17. Ion chromatogram collected from post-anolyte solution recorded for control experiment with the heterogenized **[CuT2]**$^{+}$ catalyst deposited electrode but without PFOA in the aqueous electrolyte.

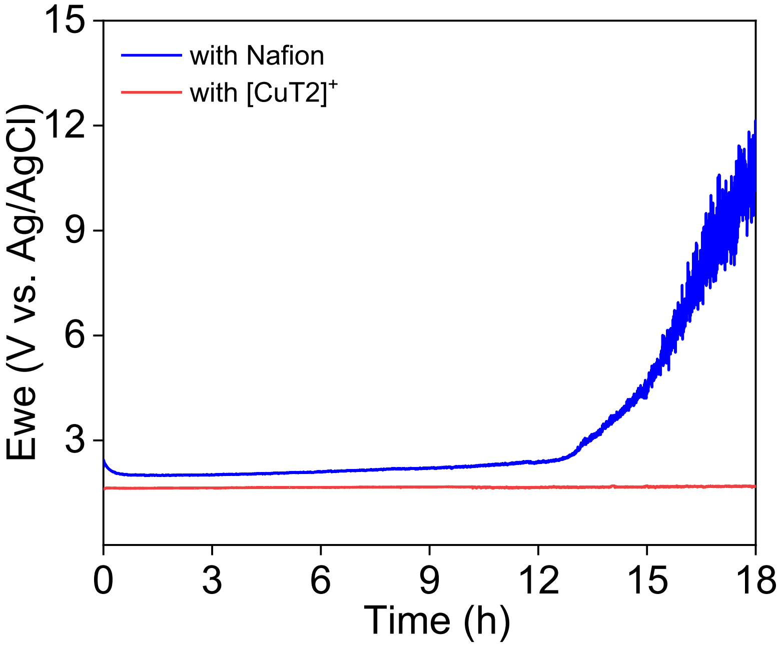

Figure S18. Working electrode potential vs. time profile recorded for PFOA oxidation during controlled-current electrolysis at 5 mA for 18 h using heterogenized **[CuT2]**$^+$ catalyst (blue) and Nafion immobilized carbon electrodes (red).

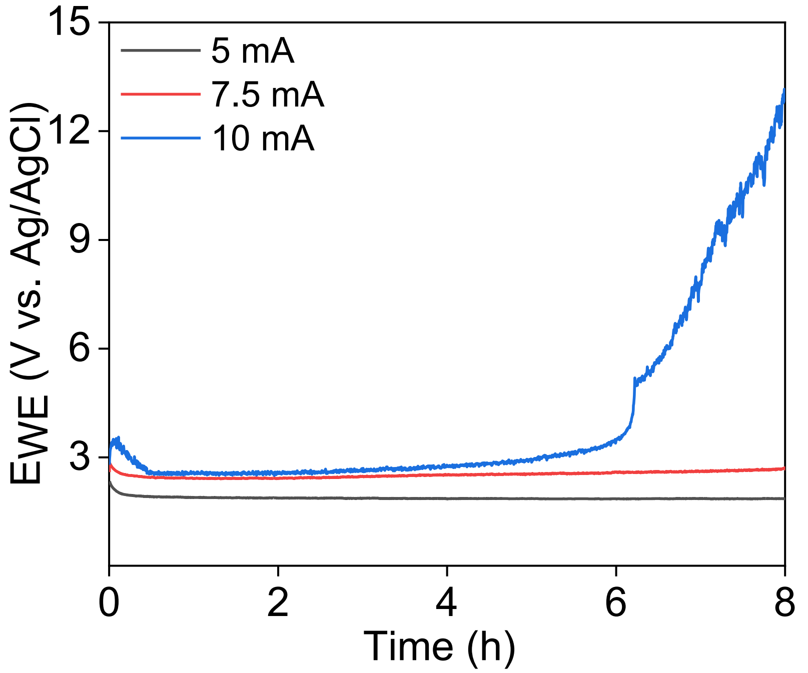

Figure S19. Controlled-current electrolysis using **[CuT2]**$^+$ adsorbed carbon paper electrodes at 5, 7.5, and 10 mA of the applied current in N_2-saturated 0.1 M $KHCO_3$ solution containing 2 mM PFOA.

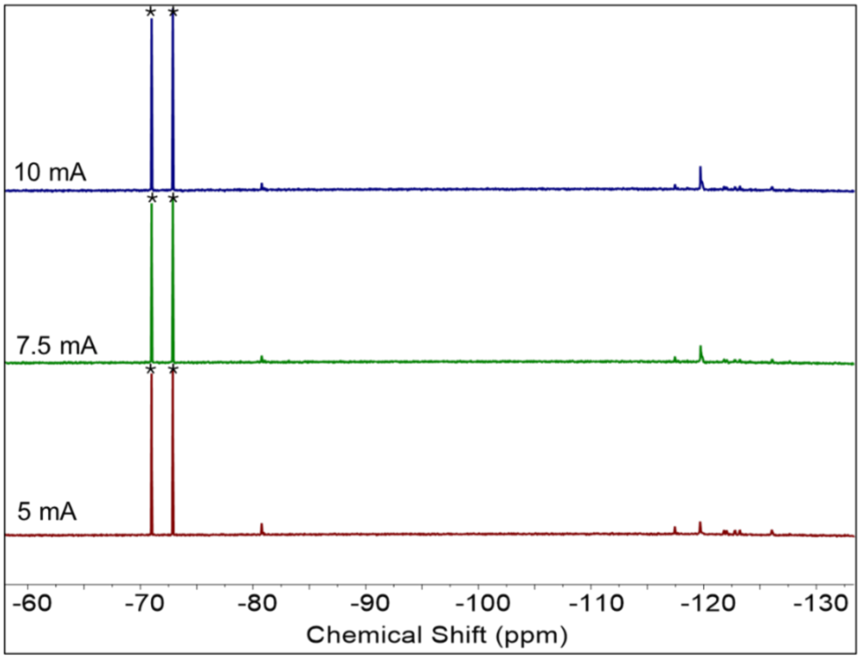

Figure S20. ^{19}F NMR spectrum recorded for post-electrolysis solution of PFOA oxidation at 5, 7.5, and 10 mA for 8 h. The asterisk symbol at peaks –71.00 and –72.88 ppm represents the internal standard, KPF_6.

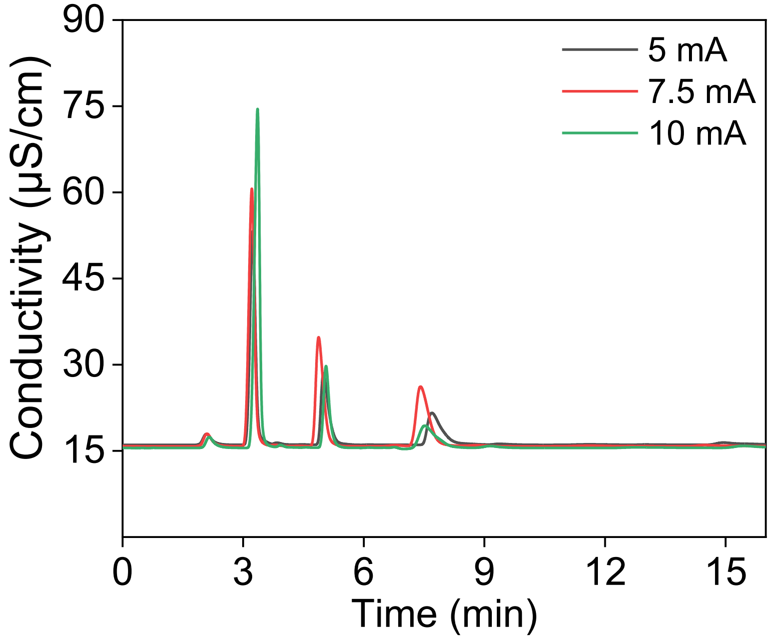

Figure S21. Ion chromatogram collected from post-controlled current electrolysis (CCE) solution of PFOA after running CCE at 5, 7.5, and 10 mA for 8 h in the 0.1M $KHCO_3$ electrolytic solution. Trace amount of chloride and nitrate ions were identified as common contaminants present in the glassware.

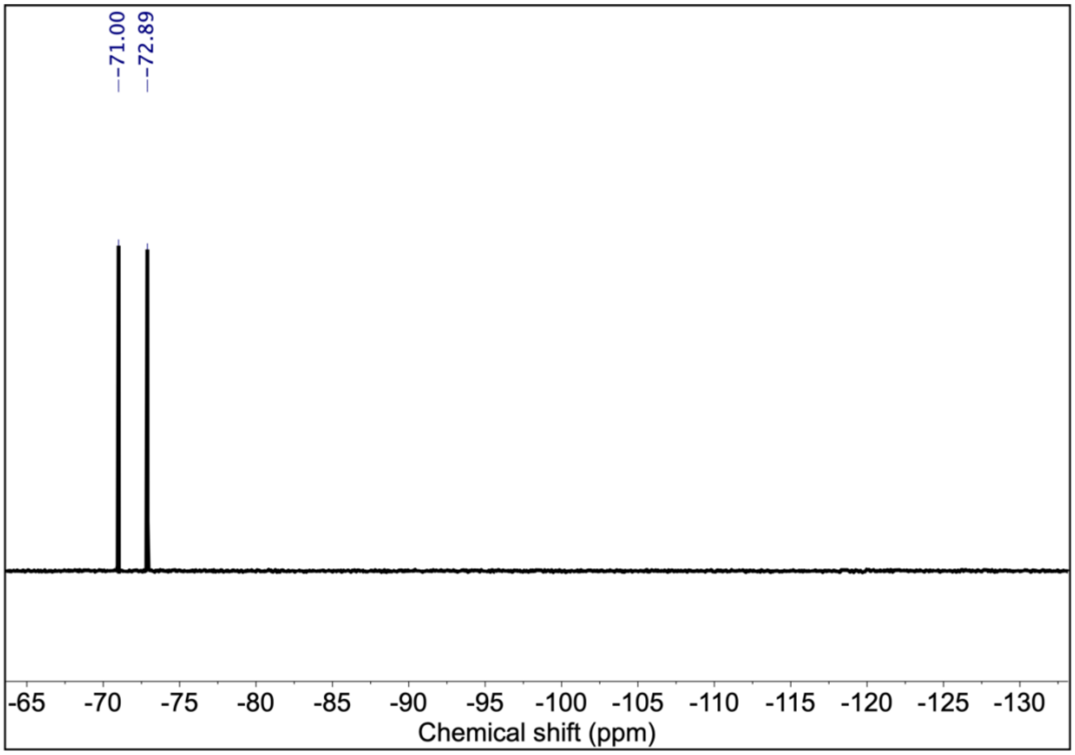

Figure S22. ^{19}F NMR spectrum recorded for post-electrolysis solution of 0.5 mM PFOA oxidation at 5 mA for 18 h. The peaks at –71.00 and –72.89 ppm are from the internal standard, KPF_6.

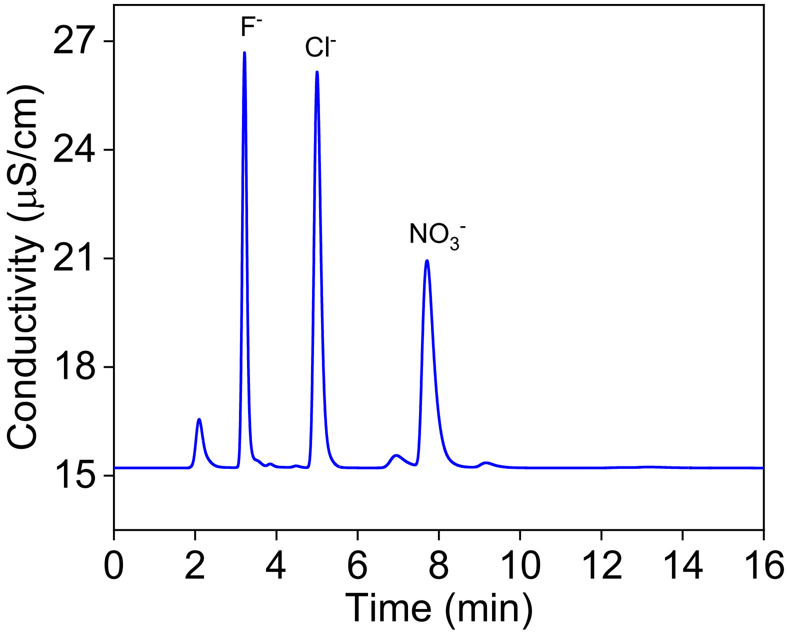

Figure S23. Ion chromatogram collected from post-controlled current electrolysis (CCE) solution after running CCE at 5 mA for 0.5 mM PFOA in the 0.1 M $KHCO_3$ electrolytic solution. Trace amount of chloride and nitrate ions were identified as common contaminants present in the glassware.

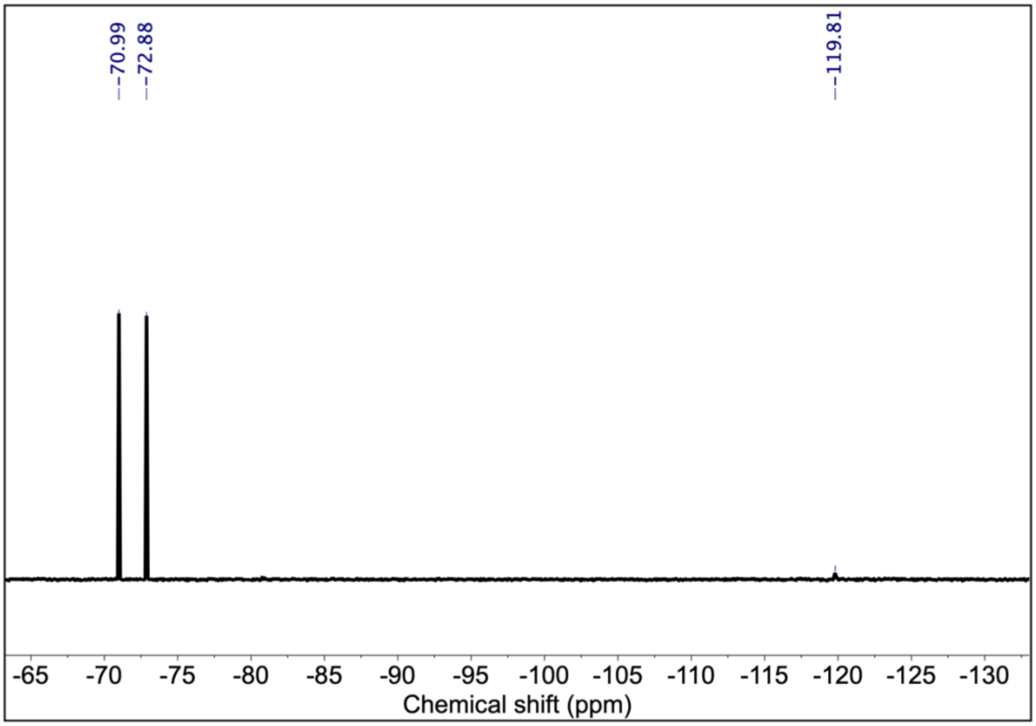

Figure S24. ^{19}F NMR spectrum recorded for post-electrolysis solution of 1 mM PFOA oxidation at 5 mA for 18 h. The peaks at –70.99 and –72.88 ppm originate from the internal standard, KPF_6.

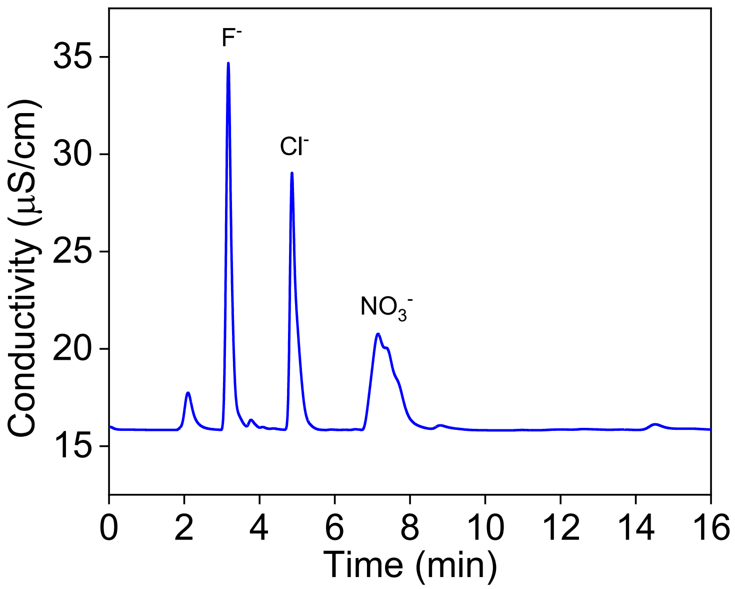

Figure S25. Ion chromatogram collected from post-controlled current electrolysis (CCE) solution after running CCE at 5 mA for 1 mM PFOA in the 0.1 M $KHCO_3$ electrolytic solution. Trace amount of chloride and nitrate ions were identified as common contaminants present in the glassware.

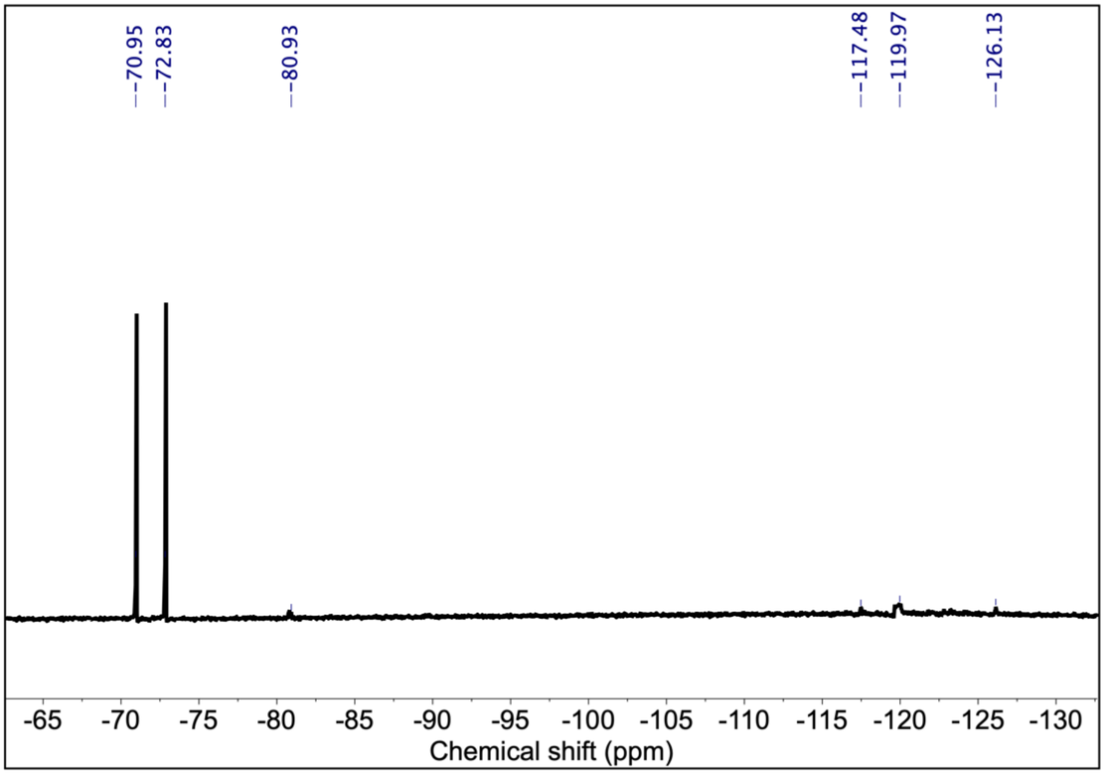

Figure S26. ^{19}F NMR spectrum recorded for post-electrolysis solution of 1.5 mM PFOA oxidation at 5 mA for 18 h. The peaks at –70.95 and –72.83 ppm originate from the internal standard, KPF_6.

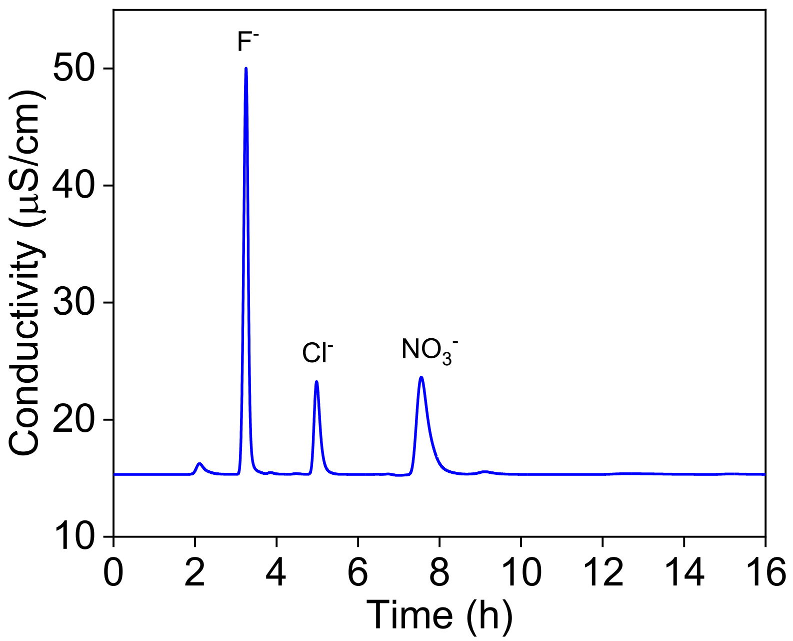

Figure S27. Ion chromatogram collected from post-controlled current electrolysis (CCE) solution after running CCE at 5 mA for 1.5 mM PFOA in the 0.1 M $KHCO_3$ electrolytic solution. Trace amount of chloride and nitrate ions were identified as common contaminants present in the glassware.

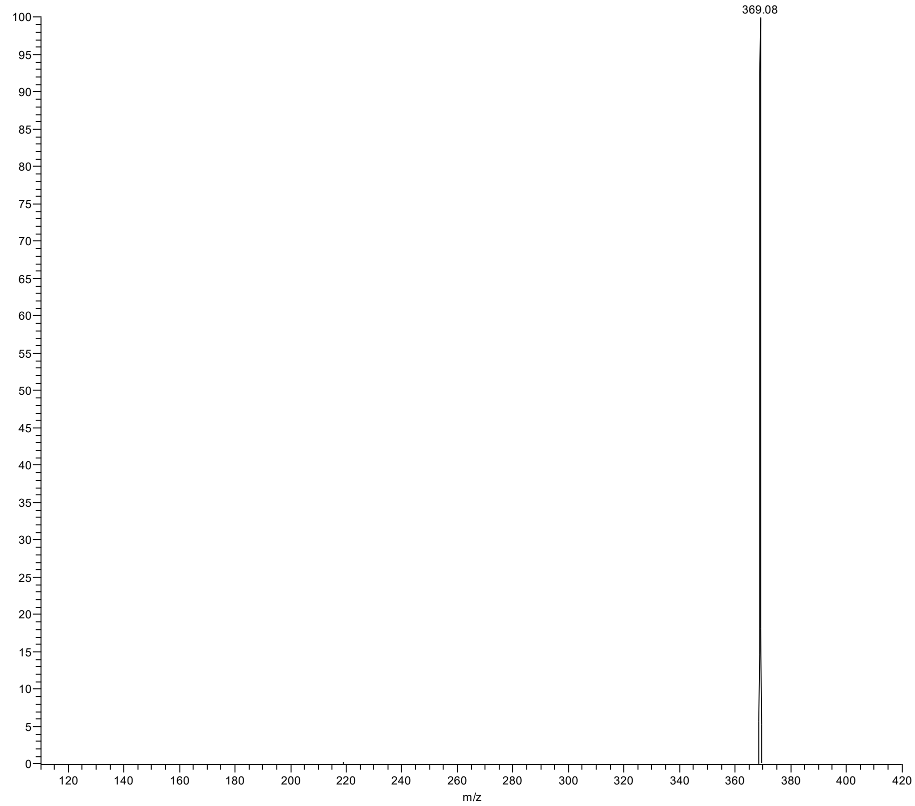

Figure S28. ESI-MS data collected for the pre-controlled-current electrolysis (CCE) solution before running CCE at 5 mA with 2 mM PFOA in N_2-saturated 0.1 M $KHCO_3$ solution. Working electrode: heterogenized **[CuT2]**$^+$ carbon paper.

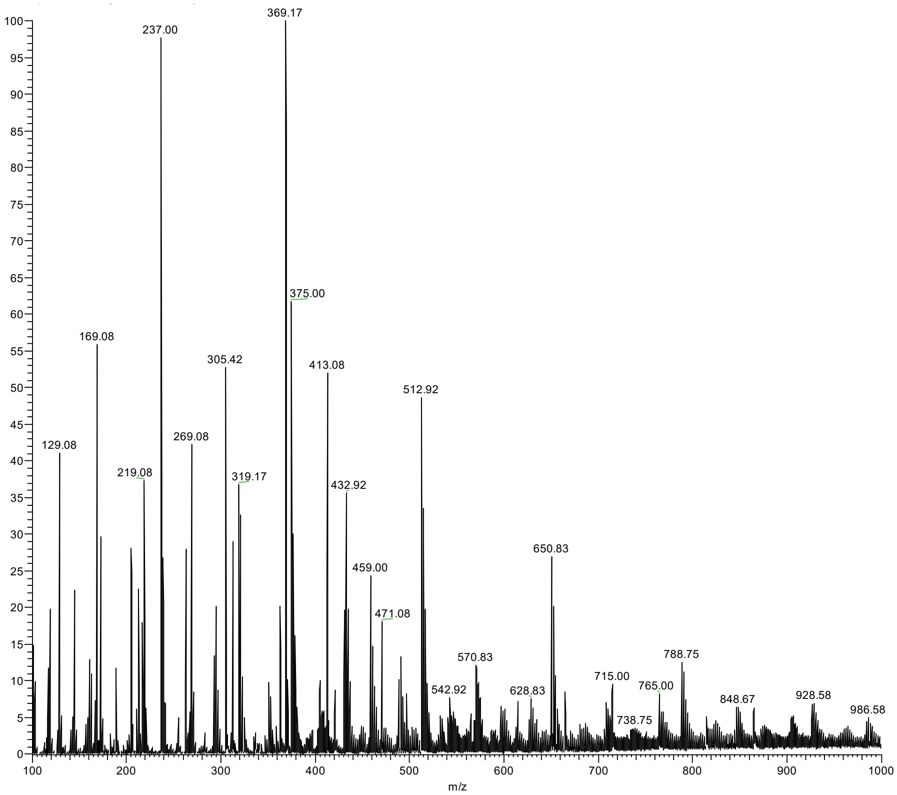

Figure S29. ESI-MS data collected for the post-controlled-current electrolysis (CCE) solution after running CCE at 5 mA with 2 mM PFOA in N_2-saturated 0.1 M $KHCO_3$ solution. Working electrode: heterogenized **[CuT2]**$^+$ carbon paper.

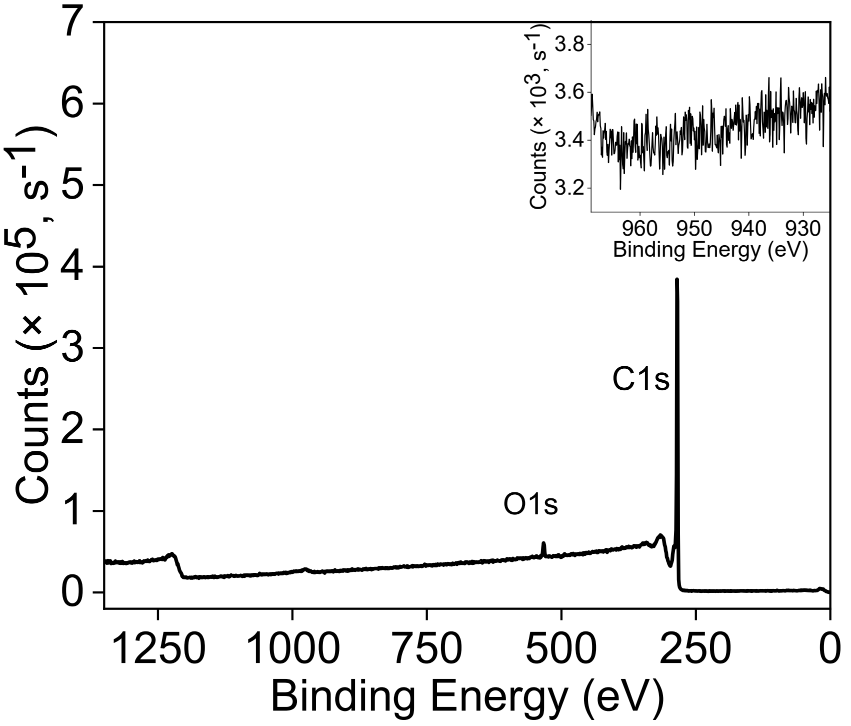

Figure S30. XPS of a bare carbon paper electrode. Inset: Expanded Cu $2p_{1/2}$ and $2p_{2/3}$ region.

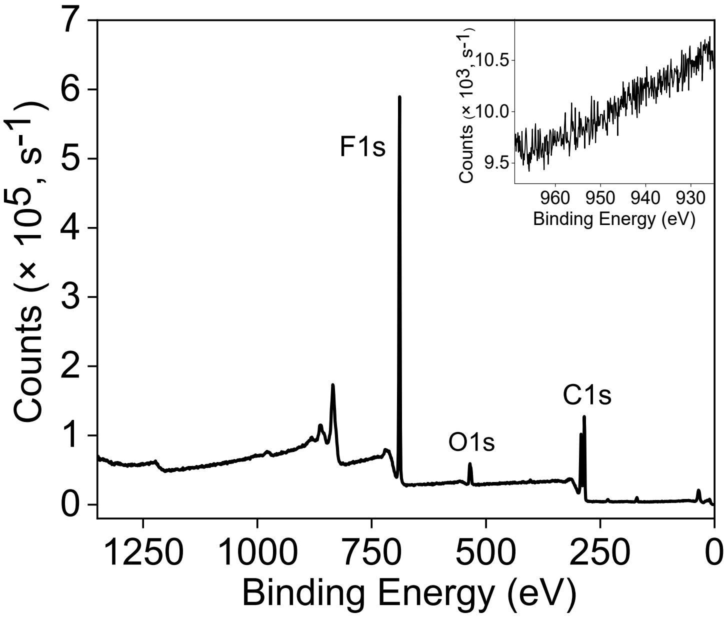

Figure S31. XPS of a rinsed **[CuT2]**$^+$-deposited carbon paper electrode. Inset: Expanded Cu $2p_{1/2}$ and $2p_{2/3}$ region.

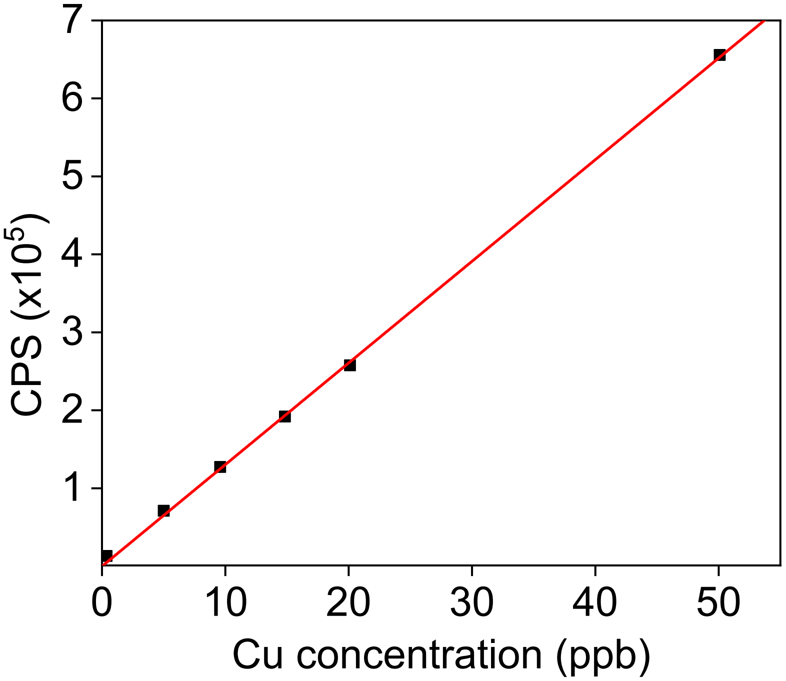

Figure S32. Inductively coupled plasma mass spectroscopy calibration curve for the determination of copper concentration.

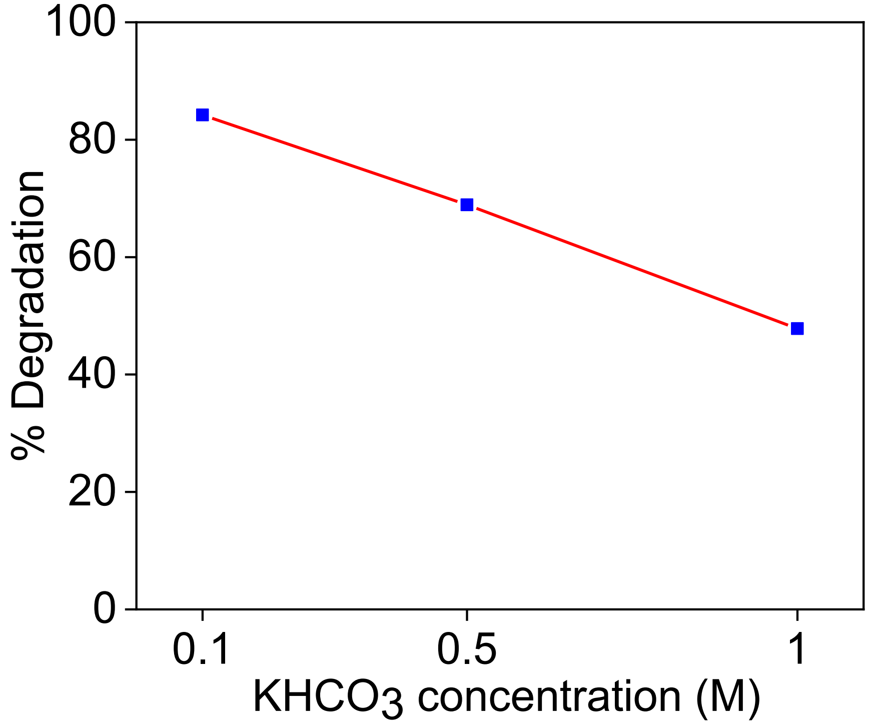

Figure S33. %PFOA degradation obtained after performing controlled-current electrolysis at 5 mA over 18 h at different $KHCO_3$ concentrations, 0.1, 0.5, and 1.0 M, by using **[CuT2]**$^+$-adsorbed carbon paper electrodes in the presence of 2 mM PFOA.

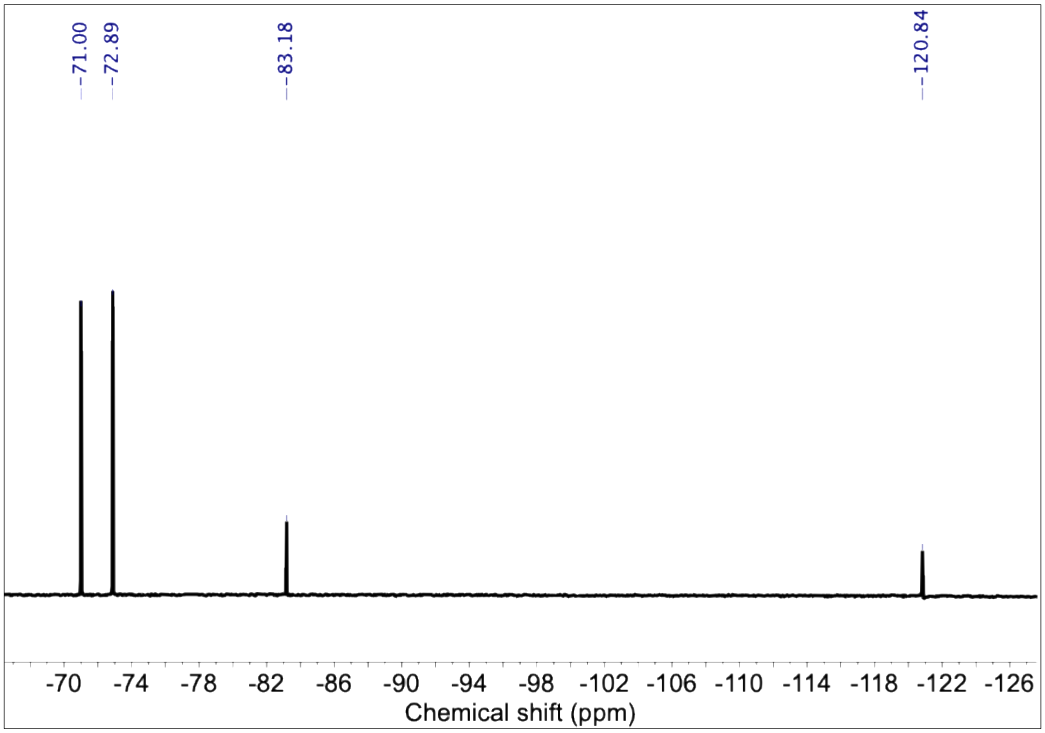

Figure S34. ^{19}F NMR spectrum recorded for pre-electrolysis solution of 2 mM pentafluoropropionic acid (PFPA) in 0.1M $KHCO_3$. The peaks at –71.00 and –72.89 ppm are from the internal standard, KPF_6.

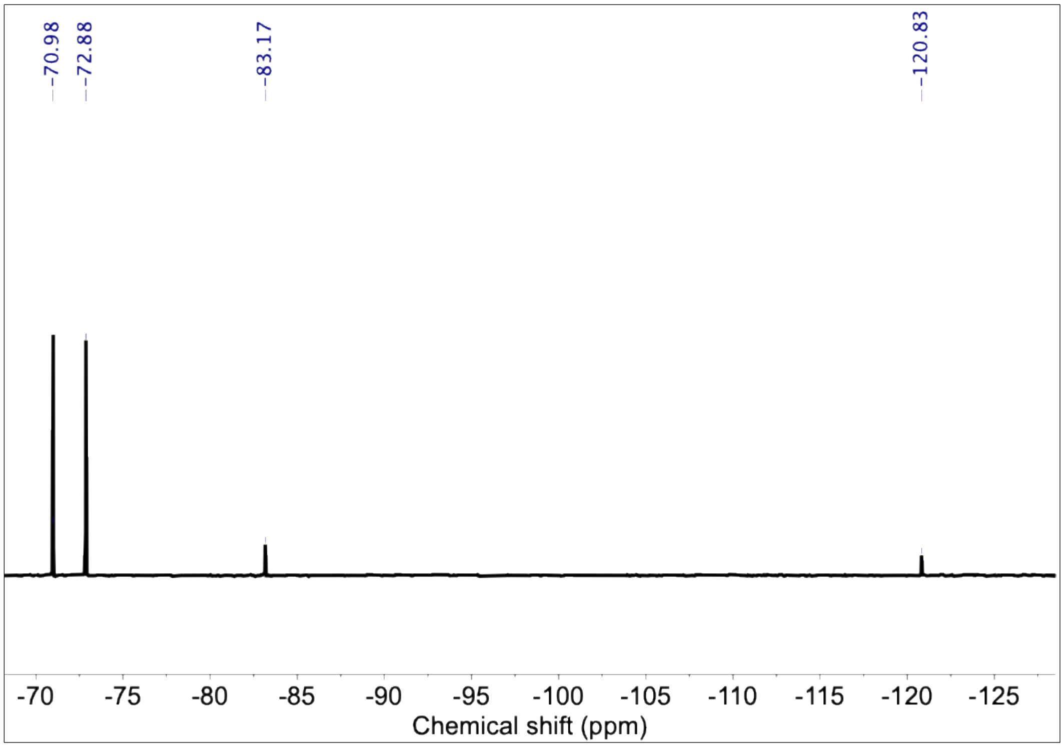

Figure S35. ^{19}F NMR spectrum recorded for post-electrolysis solution of 2 mM pentafluoropropionic acid (PFPA) oxidation at 5 mA for 18 h. The peaks at –70.98 and –72.88 ppm are from the internal standard, KPF_6.

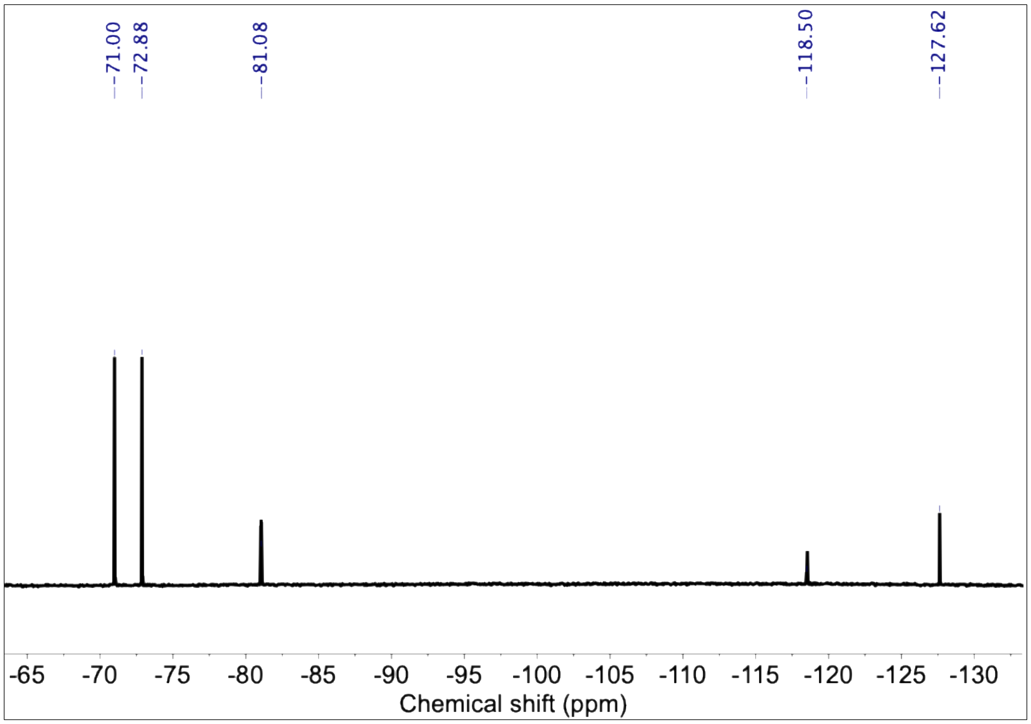

Figure S36. ^{19}F NMR spectrum recorded for pre-electrolysis solution of 2 mM perfluorobutanoic acid (PFBA) in 0.1M $KHCO_3$. The peaks at –71.00 and –72.88 ppm are from the internal standard, KPF_6.

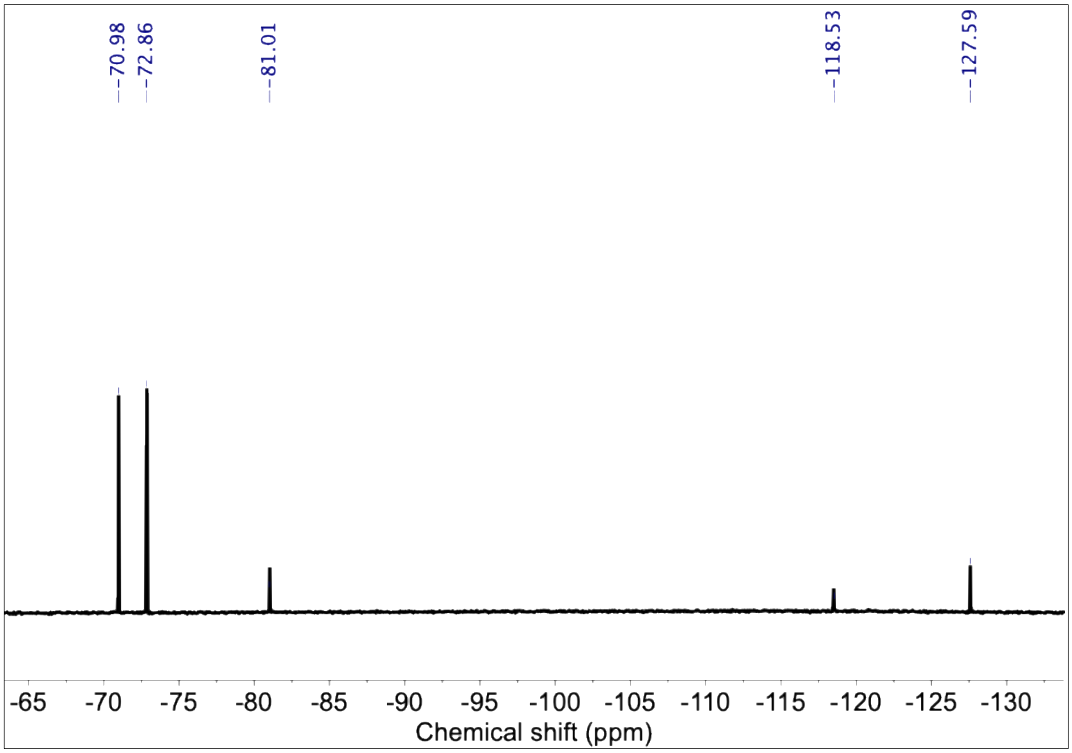

Figure S37. ^{19}F NMR spectrum recorded for post-electrolysis solution of 2 mM perfluorobutanoic acid (PFBA) oxidation at 5 mA for 18 h. The peaks at –70.98 and –72.86 ppm are from the internal standard, KPF_6.

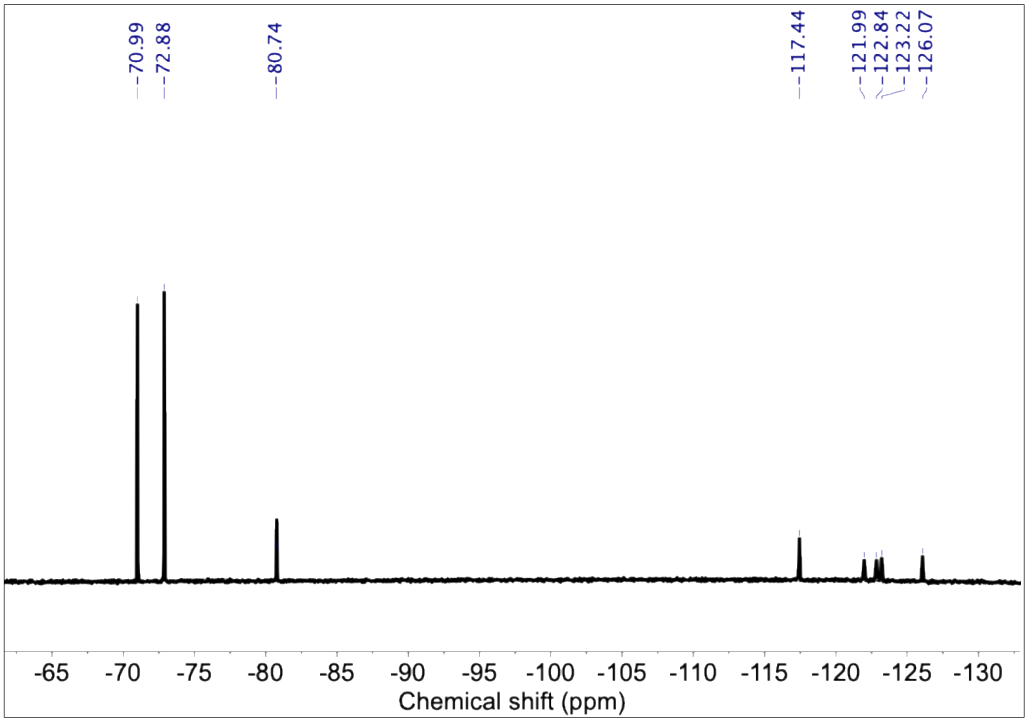

Figure S38. ^{19}F NMR spectrum recorded for pre-electrolysis solution of 2 mM perfluoroheptanoic acid (PFHA) in 0.1M $KHCO_3$. The peaks at –70.99 and –72.88 ppm are from the internal standard, KPF_6

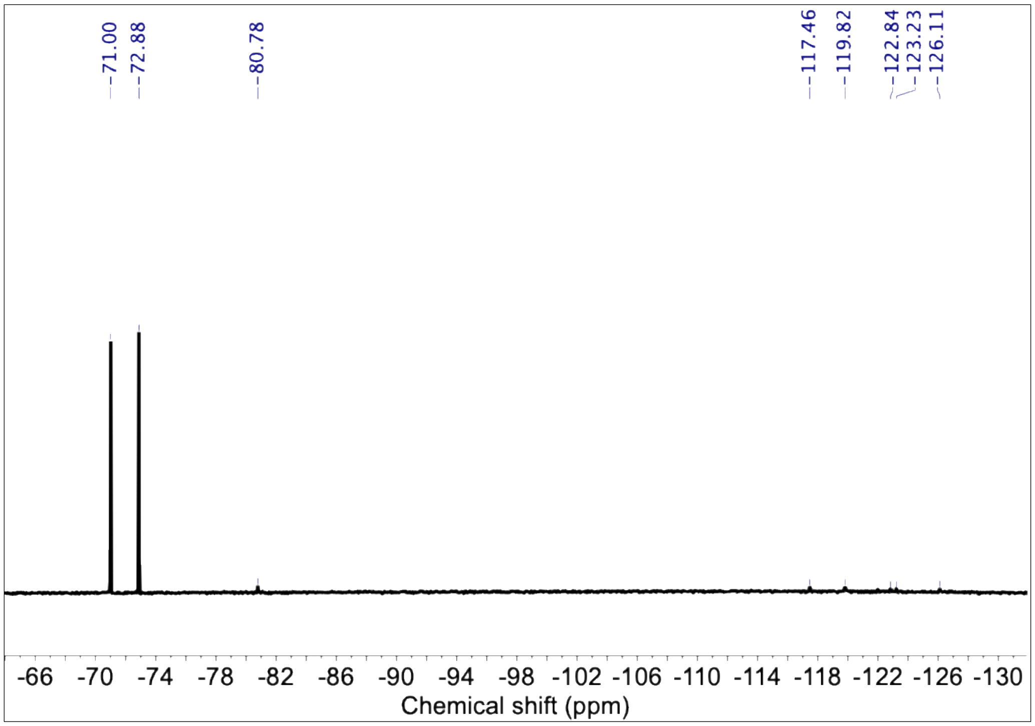

Figure S39. ^{19}F NMR spectrum recorded for post-electrolysis solution of 2 mM perfluoroheptanoic acid (PFHA) oxidation at 5 mA for 18 h. The peaks at –71.00 and –72.88 ppm are from the internal standard, KPF_6.

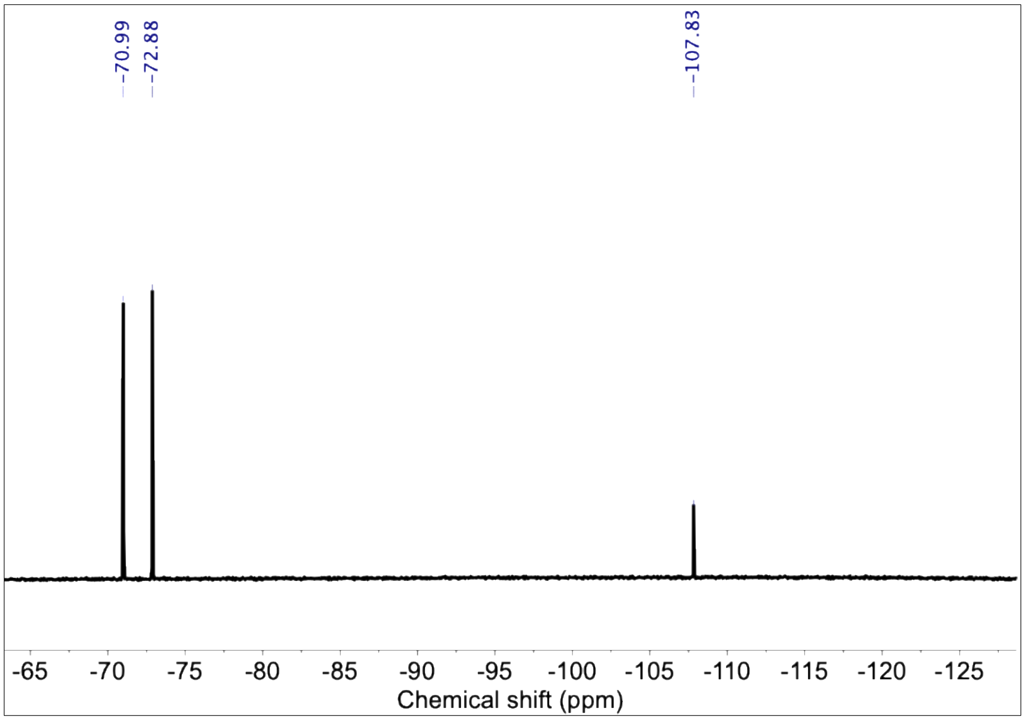

Figure S40. ^{19}F NMR spectrum recorded for pre-electrolysis solution of 2 mM difluoropropanedioic acid (DFPDA) in 0.1M $KHCO_3$. The peaks at –70.99 and –72.88 ppm are from the internal standard, KPF_6.

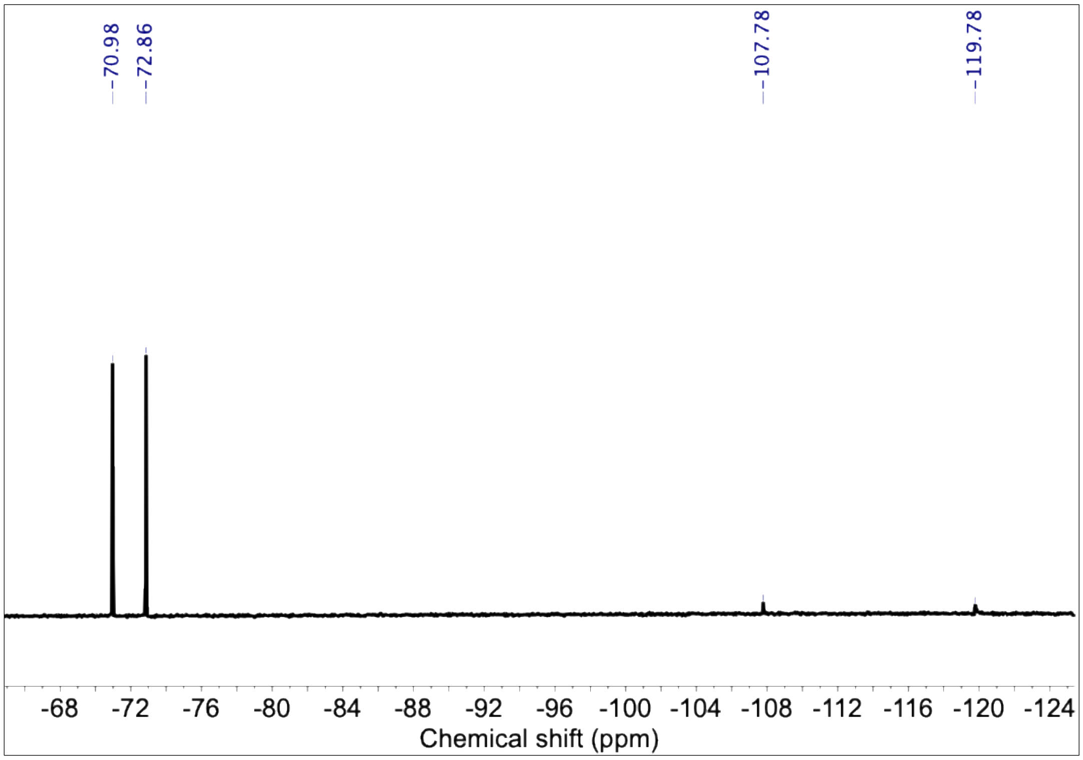

Figure S41. ^{19}F NMR spectrum recorded for post-electrolysis solution of 2 mM difluoropropanedioic acid (DFPDA) oxidation at 5 mA for 18 h. The peaks at –70.98 and –72.86 ppm are from the internal standard, KPF_6.

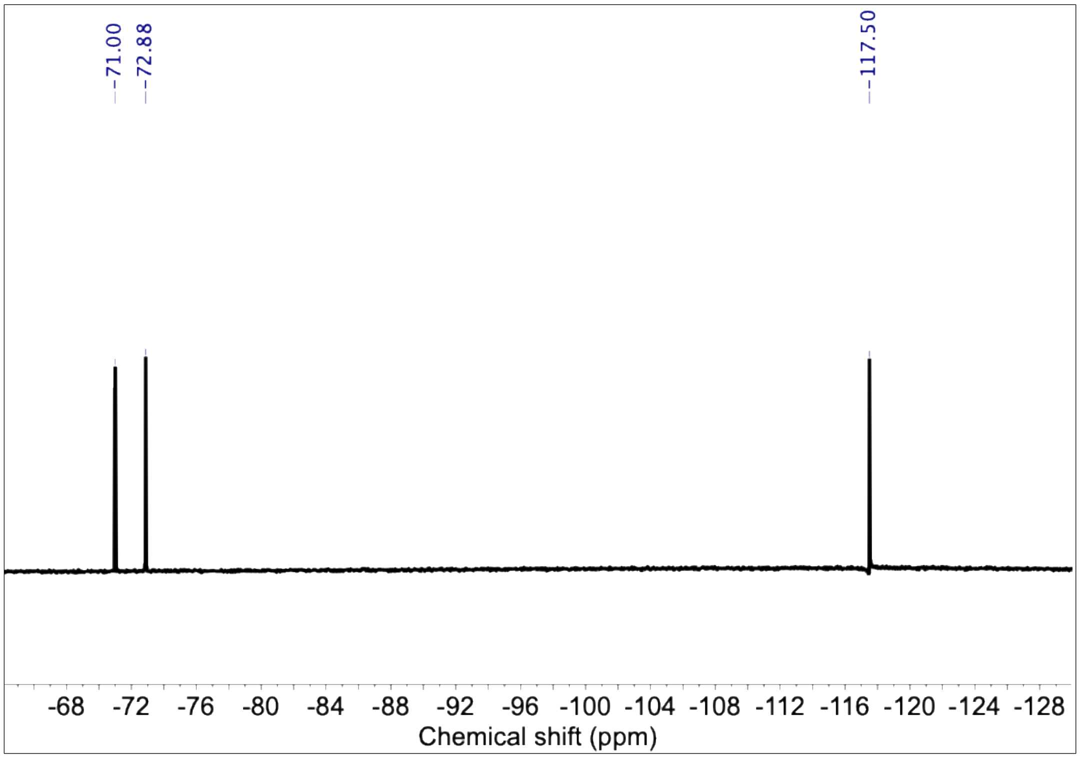

Figure S42. ^{19}F NMR spectrum recorded for pre-electrolysis solution of 2 mM tetrafluorobutanedioic acid (TFBDA) in 0.1M $KHCO_3$. The peaks at –71.00 and –72.88 ppm are from the internal standard, KPF_6.

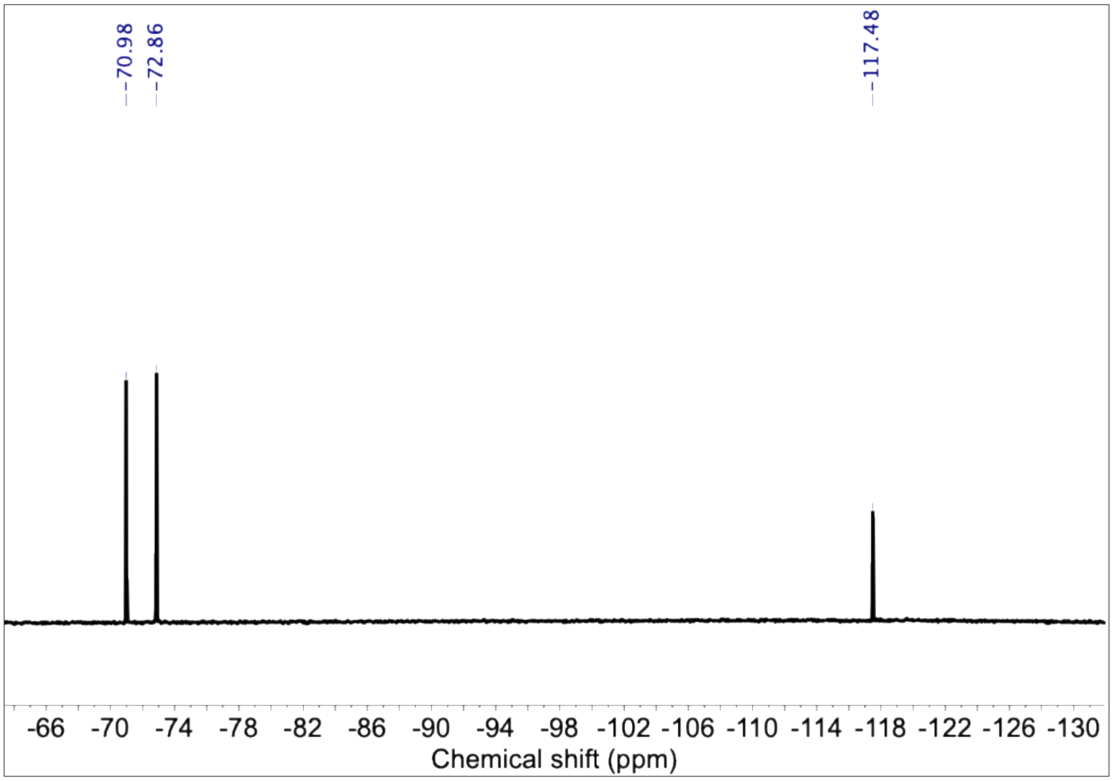

Figure S43. ^{19}F NMR spectrum recorded for post-electrolysis solution of 2 mM tetrafluorobutanedioic acid (TFBDA) oxidation at 5 mA for 18 h. The peaks at –70.98 and –72.86 ppm are from the internal standard, KPF_6

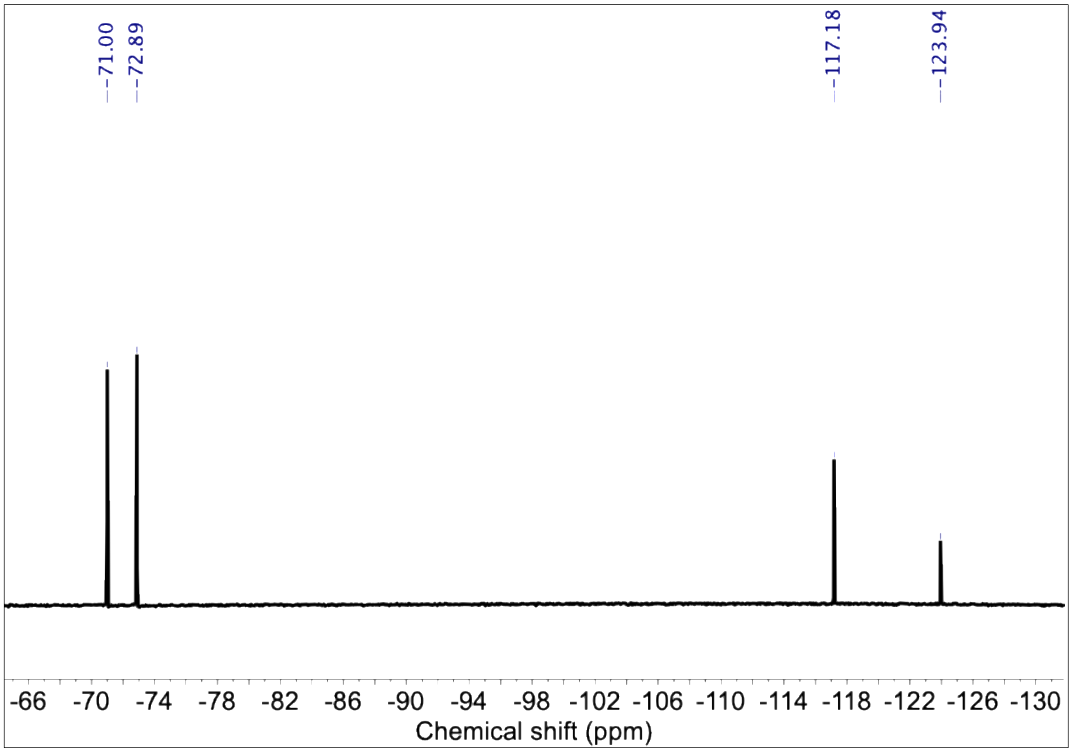

Figure S44. ^{19}F NMR spectrum recorded for pre-electrolysis solution of 2 mM hexafluoropentanedioic acid (HFPDA) in 0.1 M $KHCO_3$. The peaks at –71.00 and –72.89 ppm are from the internal standard, KPF_6

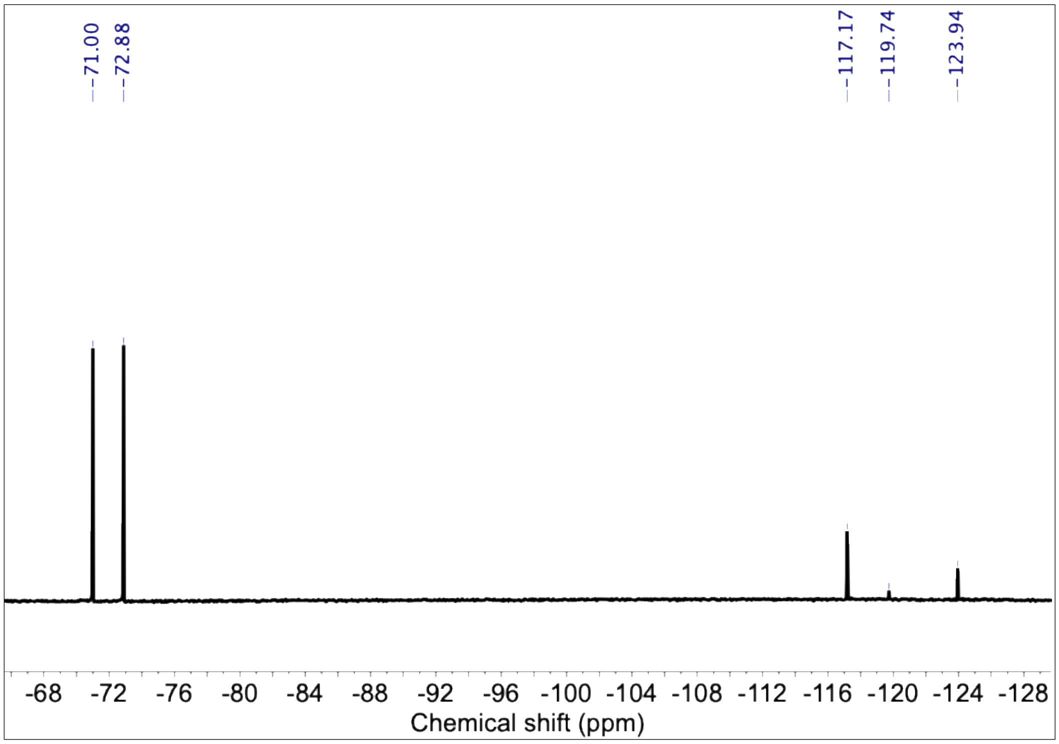

Figure S45. ^{19}F NMR spectrum recorded for post-electrolysis solution of 2 mM hexafluoropentanedioic acid (HFPDA) oxidation at 5 mA for 18 h. The peaks at –71.00 and –72.88 ppm are from the internal standard, KPF_6.

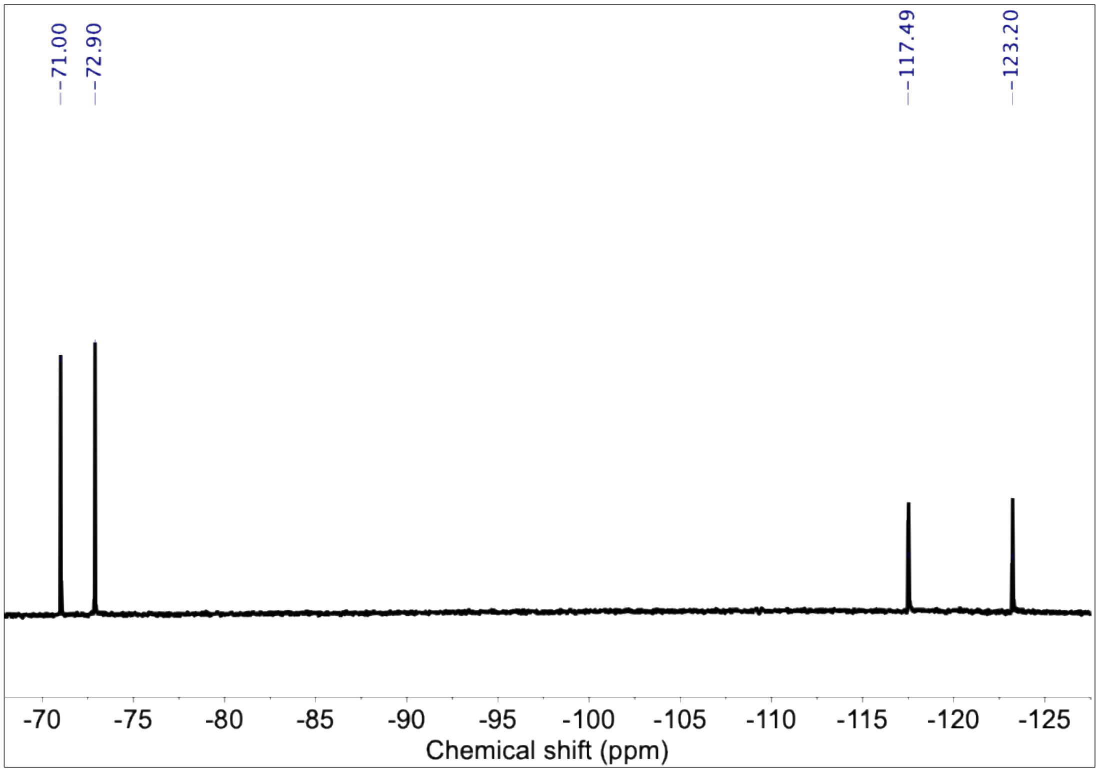

Figure S46. ^{19}F NMR spectrum recorded for pre-electrolysis solution of 2 mM octafluorohexanedioic acid (OFHDA) in 0.1 M $KHCO_3$. The peaks at –71.00 and –72.90 ppm are from the internal standard, KPF_6.

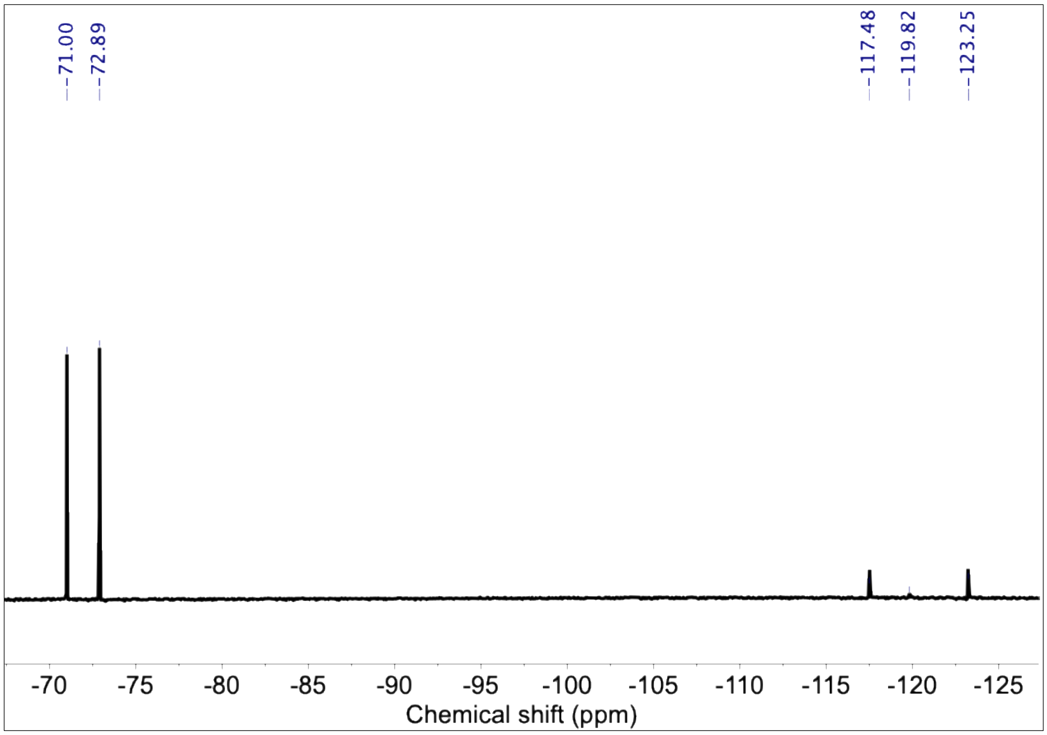

Figure S47. ^{19}F NMR spectrum recorded for post-electrolysis solution of 2 mM octafluorohexanedioic acid (OFHDA) oxidation at 5 mA for 18 h. The peaks at –71.00 and –72.89 ppm are from the internal standard, KPF_6.

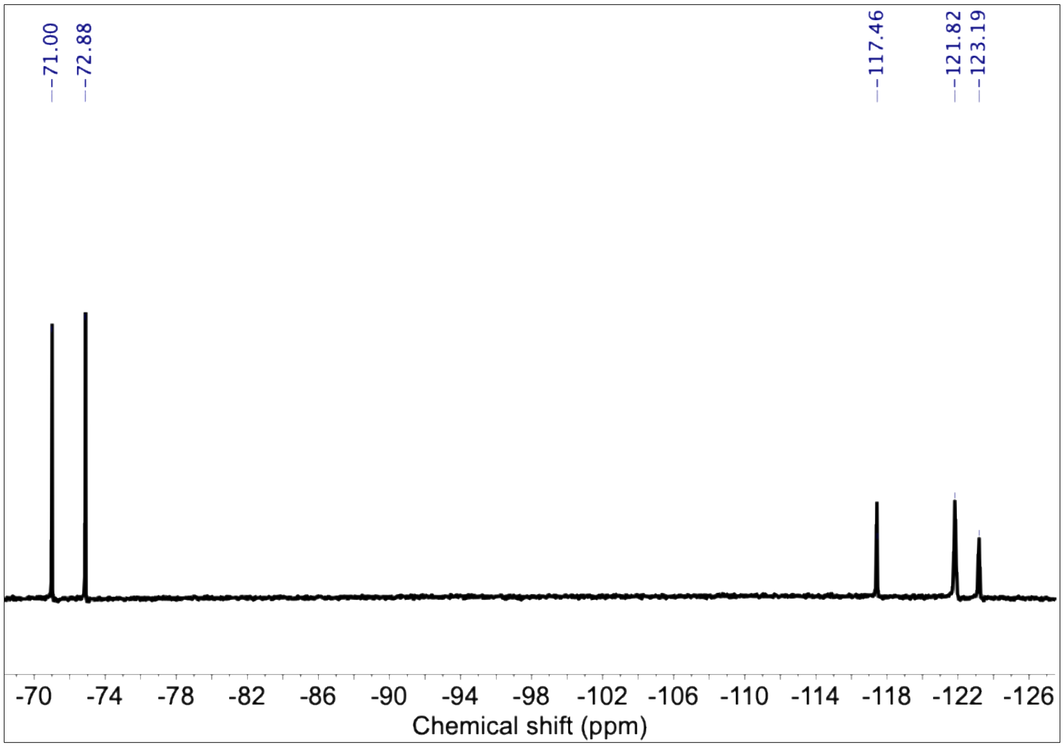

Figure S48. ^{19}F NMR spectrum recorded for pre-electrolysis solution of 2 mM hexadecafluorodecanedioic acid (HDFDA) in 0.1 M $KHCO_3$. The peaks at –71.00 and –72.88 ppm are from the internal standard, KPF_6.

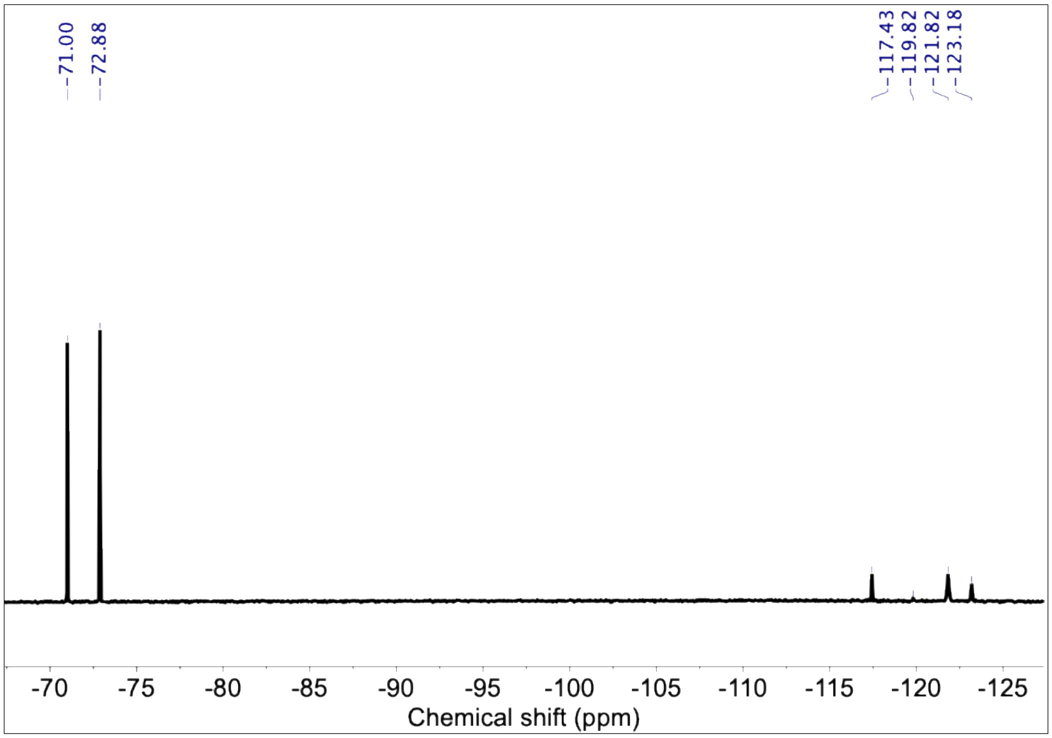

Figure S49. ^{19}F NMR spectrum recorded for post-electrolysis solution of 2 mM hexadecafluordecanedioic acid (HDFDA) oxidation at 5 mA for 18 h. The peaks at –71.00 and –72.88 ppm are from the internal standard, KPF_6.

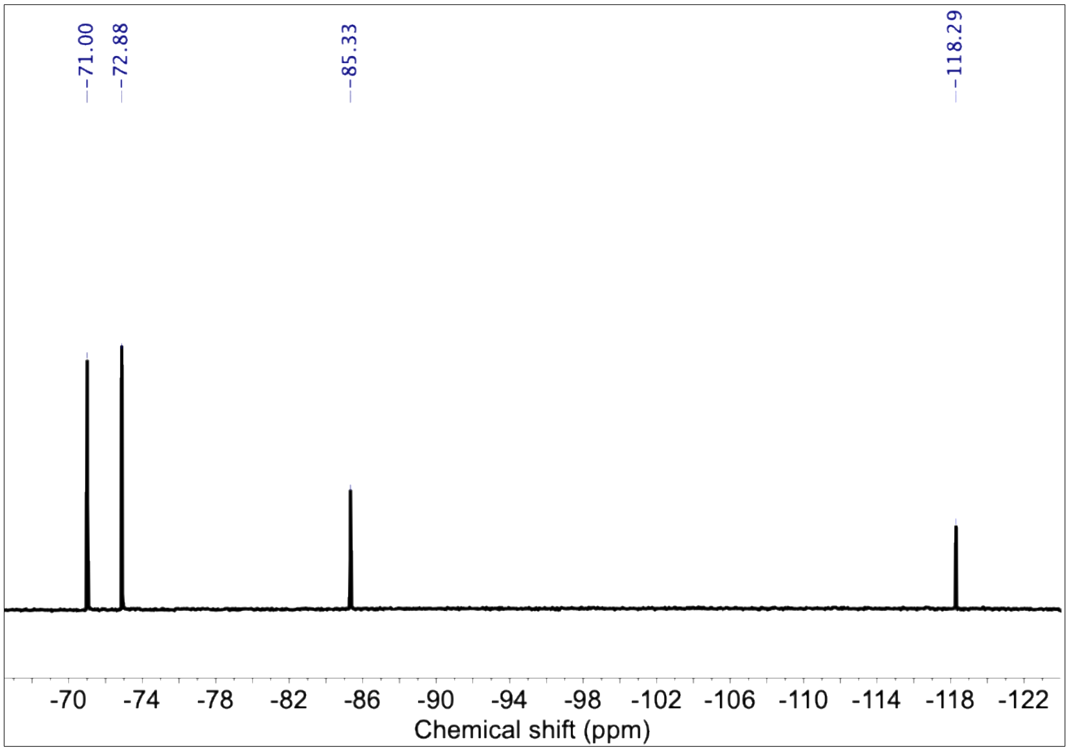

Figure S50. ^{19}F NMR spectrum recorded for pre-electrolysis solution of 2 mM perfluoropentanoic acid (PFPC2A) in 0.1 M $KHCO_3$. The peaks at –71.00 and –72.88 ppm are from the internal standard, KPF_6.

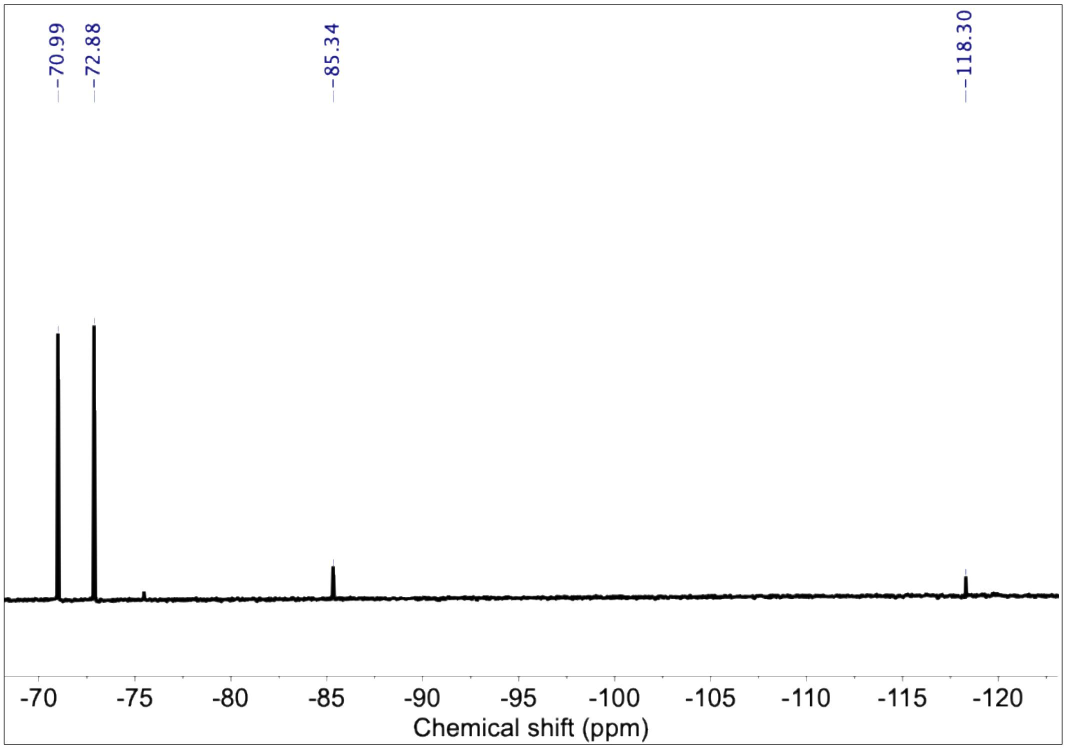

Figure S51. ^{19}F NMR spectrum recorded for post-electrolysis solution of 2 mM perfluoropentanoic acid (PFPC2A) oxidation at 5 mA for 18 h. The peaks at –70.99 and –72.88 ppm are from the internal standard, KPF_6.

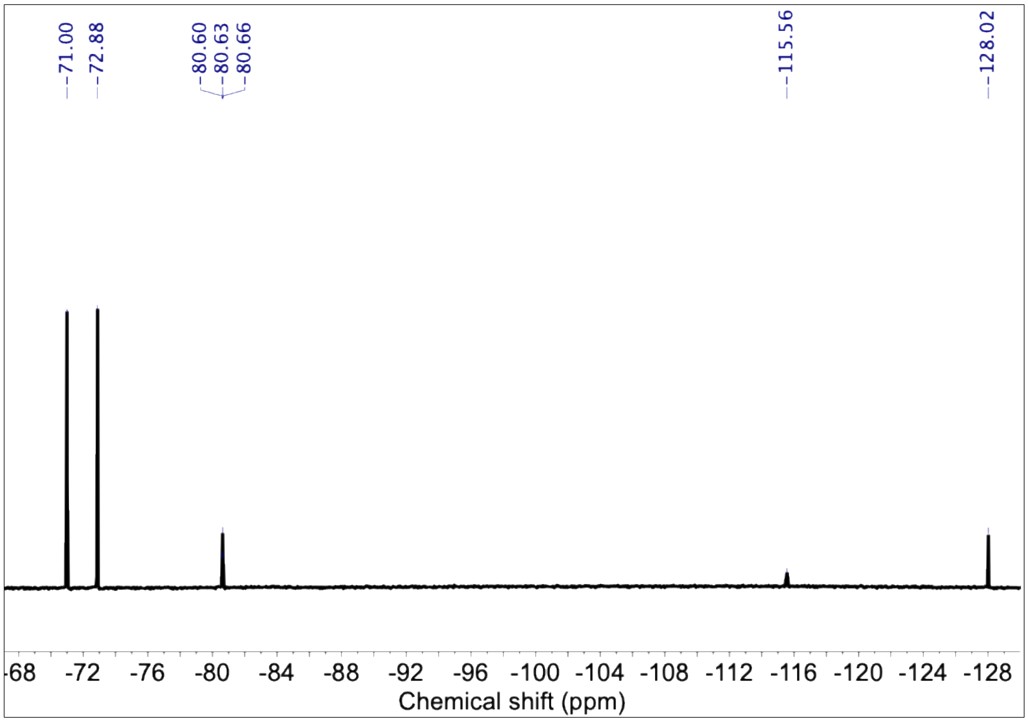

Figure S52. ^{19}F NMR spectrum recorded for pre-electrolysis solution of 2 mM heptafluorohexanoic acid (HFHC2A) in 0.1M $KHCO_3$. The peaks at –71.00 and –72.88 ppm are from the internal standard, KPF_6.

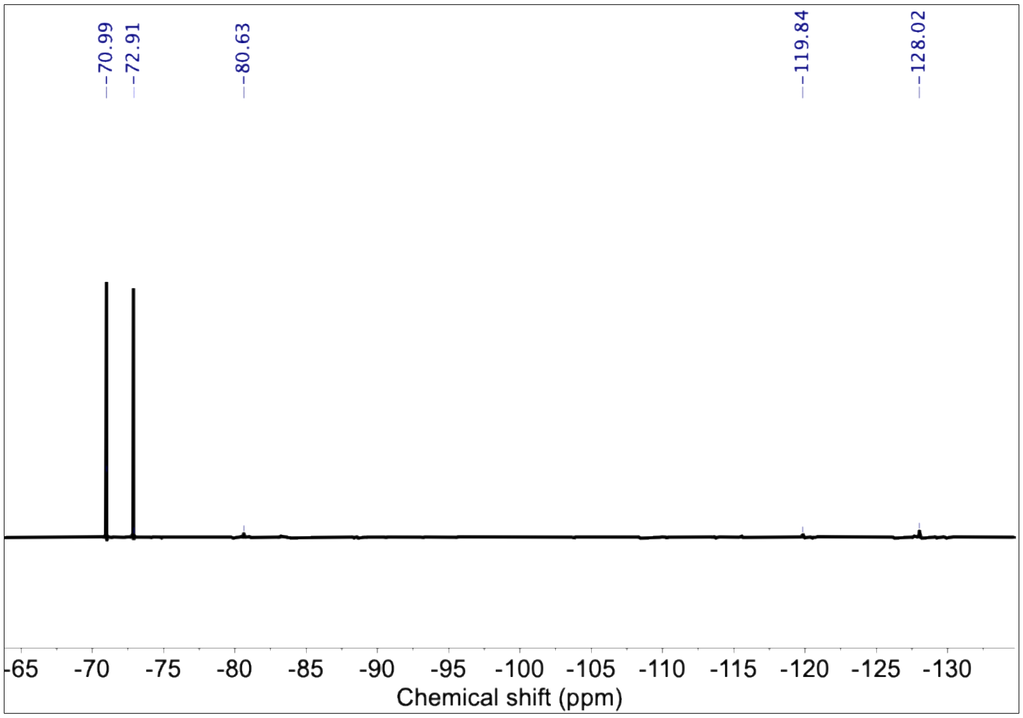

Figure S53. ^{19}F NMR spectrum recorded for post-electrolysis solution of 2 mM heptafluorohexanoic acid (HFHC2A) oxidation at 5 mA for 18 h. The peaks at –70.99 and –72.91 ppm are from the internal standard, KPF_6.

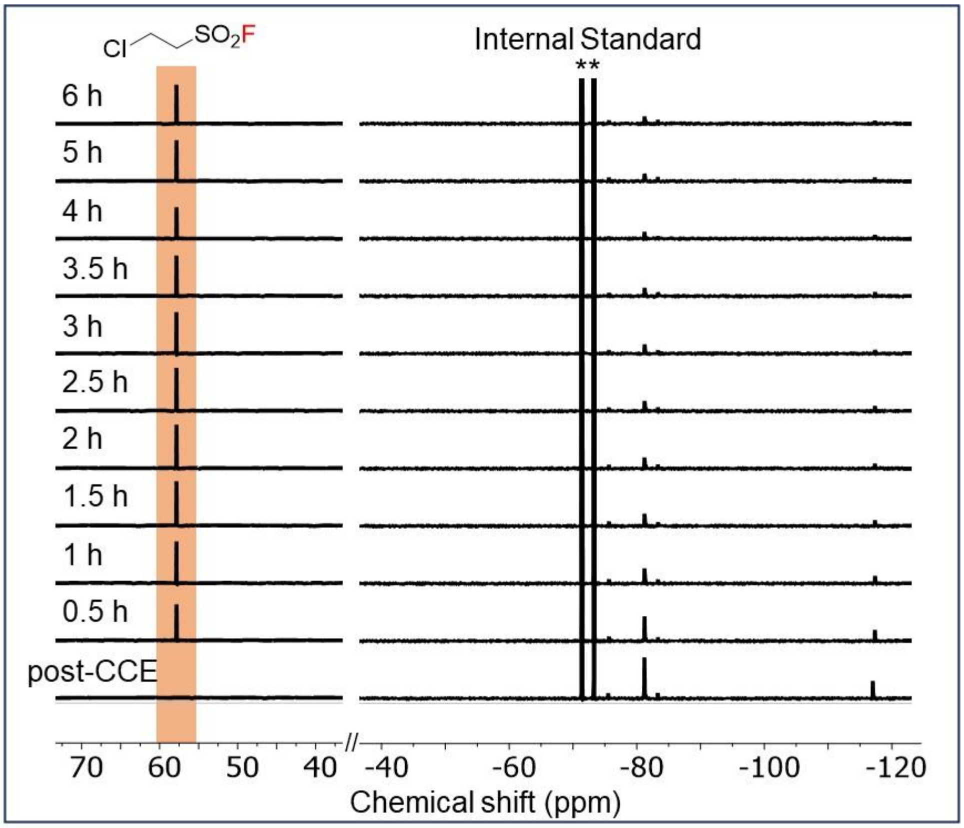

Figure S54. Time-dependent ^{19}F NMR spectra recorded for post-CCE solution while stirring the solution upon adding $Cl(CH_2)_2SO_2Cl$ without applying current. The peaks at –71.44 and –73.29 ppm are from the internal standard, $TBAPF_6$.

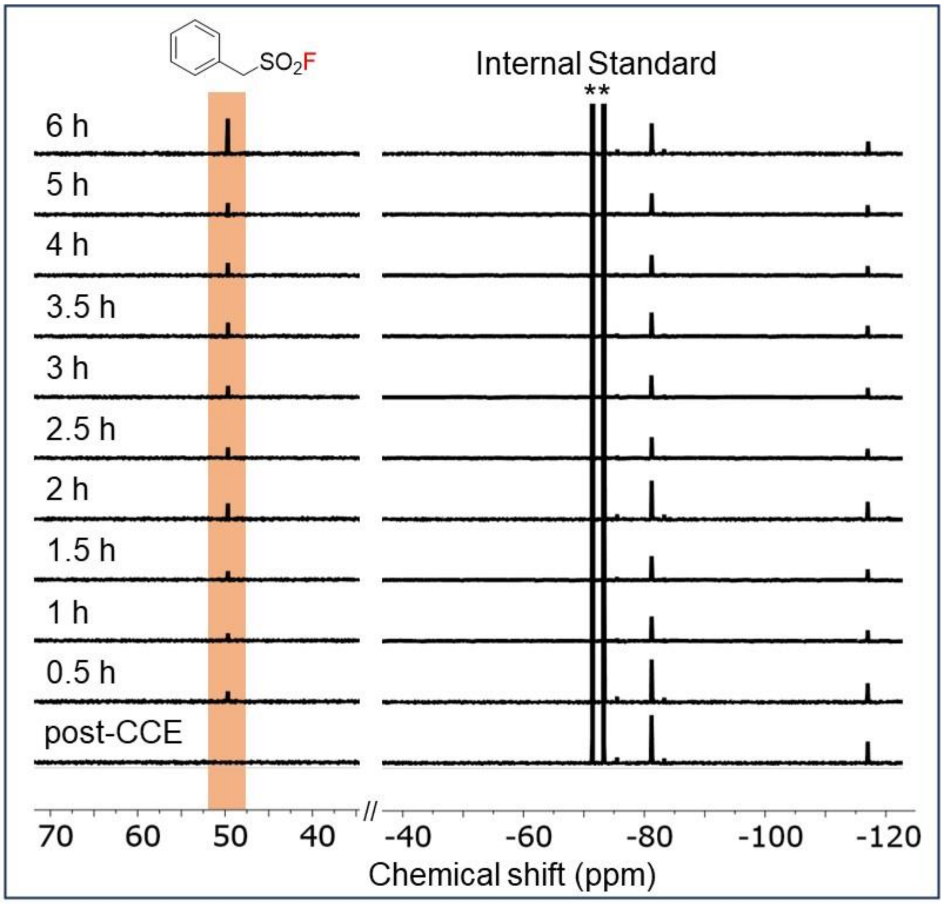

Figure S55. Time-dependent ^{19}F NMR spectra recorded for post-CCE solution while stirring the solution upon adding $BzSO_2Cl$ without applying current. The peaks at –71.44 and –73.29 ppm are from the internal standard, $TBAPF_6$.

Table S1. Atomic percentage derived from the XPS survey scans.

Peak	Fresh Electrode	Used Electrode
Cu2p	0.82%	0.60%
N1s	5.1%	5.68
O1s	6.33%	10.47
C1s	48.79%	35.28
F1s	37.43%	45.19%

Table S2. Energy consumption of PFOA degradation using different reported methods.

PFAS degradation method	Energy Consumption ($kWhm^{-3}$)	Reference
Sonochemical (354 kHz)	4045	1
Photocatalysis (UV/TiO_2-Oxalic acid)	158	2
UV/KI	95	3
Electrochemical Oxidation (nanocrystalline boron-doped diamond anode)	180	4
Electrochemical oxidation (Ti-SnO_2-Sb-Bi)	265	5
Heterogenized **[CuT2]**$^+$ under electro-oxidation	86.2	this work

Table S3. PFOA degradation and defluorination obtained for different PFAS substrates.

PFAS Substrates	PFAS Degradation (%)	Fluoride Recovery (%)
PFPA	34	4
PFBA	40	23
PFHA	87	40
DFPDA	81	73
TFBDA	40	41
HFPDA	44	43
OFHDA	46	46
HDFDA	48	10
PFPC2A	77	13
HFHC2A	90	18

References:

(1) Moriwaki, H.; Takagi, Y.; Tanaka, M.; Tsuruho, K.; Okitsu, K.; Maeda, Y. Sonochemical Decomposition of Perfluorooctane Sulfonate and Perfluorooctanoic Acid. *Environ. Sci. Technol.* **2005**, *39* (9), 3388–3392. https://doi.org/10.1021/es040342v.

(2) Wang, Y.; Zhang, P. Photocatalytic Decomposition of Perfluorooctanoic Acid (PFOA) by TiO2 in the Presence of Oxalic Acid. *J. Hazard. Mater.* **2011**, *192* (3), 1869–1875. https://doi.org/10.1016/j.jhazmat.2011.07.026.

(3) Qu, Y.; Zhang, C.; Li, F.; Chen, J.; Zhou, Q. Photo-Reductive Defluorination of Perfluorooctanoic Acid in Water. *Water Res.* **2010**, *44* (9), 2939–2947. https://doi.org/10.1016/j.watres.2010.02.019.

(4) Schaefer, C. E.; Andaya, C.; Burant, A.; Condee, C. W.; Urtiaga, A.; Strathmann, T. J.; Higgins, C. P. Electrochemical Treatment of Perfluorooctanoic Acid and Perfluorooctane Sulfonate: Insights into Mechanisms and Application to Groundwater Treatment. *Chem. Eng. J.* **2017**, *317*, 424–432. https://doi.org/10.1016/j.cej.2017.02.107.

(5) Zhuo, Q.; Deng, S.; Yang, B.; Huang, J.; Yu, G. Efficient Electrochemical Oxidation of Perfluorooctanoate Using a Ti/SnO_2 -Sb-Bi Anode. *Environ. Sci. Technol.* **2011**, *45* (7), 2973–2979. https://doi.org/10.1021/es1024542.
